# Clickable Polymer Ligand-Functionalized Iron Oxide Nanocubes: A Promising Nanoplatform for ‘Local Hot Spots’ Magnetically Triggered Drug Release

**DOI:** 10.1021/acsami.2c14752

**Published:** 2022-10-18

**Authors:** Binh T. Mai, John S. Conteh, Helena Gavilán, Alessandro Di Girolamo, Teresa Pellegrino

**Affiliations:** Istituto Italiano di Tecnologia, via Morego 30, 16163 Genova, Italy

**Keywords:** multifunctional polymer, heat-mediated release, magnetic hyperthermia, iron oxide nanoparticles, drug release, hot-spot effect

## Abstract

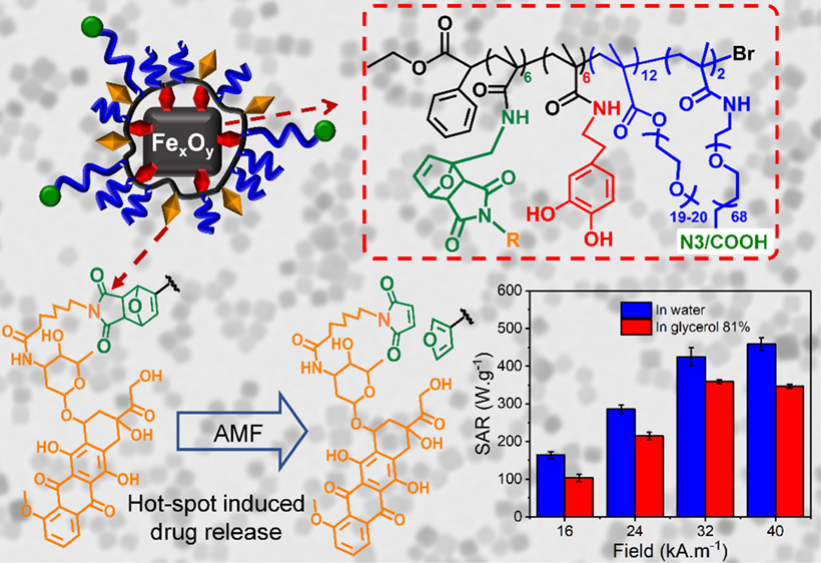

Exploiting the local heat on the surface of magnetic nanoparticles (MNPs) upon exposure to an alternating magnetic field (AMF) to cleave thermal labile bonds represents an interesting approach in the context of remotely triggered drug delivery. Here, taking advantages of a simple and scalable two-step ligand exchange reaction, we have prepared iron oxide nanocubes (IONCs) functionalized with a novel multifunctional polymer ligand having multiple catechol moieties, furfuryl pendants, and polyethylene glycol (PEG) side chains. Catechol groups ensure a strong binding of the polymer ligands to the IONCs surface, while the PEG chains provide good colloidal stability to the polymer-coated IONCs. More importantly, furfuryl pendants on the polymer enable to click the molecules of interest (either maleimide–fluorescein or maleimide–doxorubicin) via a thermal labile Diels–Alder adduct. The resulting IONCs functionalized with a fluorescein/doxorubicin-conjugated polymer ligand exhibit good colloidal stability in buffer saline and serum solution along with outstanding heating performance in aqueous solution or even in viscous media (81% glycerol/water) when exposed to the AMF of clinical use. The release of conjugated bioactive molecules such as fluorescein and doxorubicin could be boosted by applying AMF conditions of clinical use (16 kAm^–1^ and 110 kHz). It is remarkable that the magnetic hyperthermia-mediated release of the dye/drug falls in the concentration range 1.0–5.0 μM at an IONCs dose as low as 0.5 g_Fe_/L and at no macroscopical temperature change. This local release effect makes this magnetic nanoplatform a potential tool for drug delivery with remote magnetic hyperthermia actuation and with a dose-independent action of MNPs.

## Introduction

Magnetic nanoparticles (MNPs) hold tremendous potentials for diagnostic and therapeutic applications. Having unique magnetic properties, MNPs were extensively exploited as magnetic resonance imaging (MRI) contrast agents and smart drug delivery systems.^1−5^ More recently, the emerging applications of MNPs include their use as tracers in magnetic particle imaging (MPI) and as heat agents in magnetic hyperthermia (MHT) for cancer diagnosis and therapy.^6−8^ MHT relies on the exploitation of MNPs as magneto-heat transducers to convert the energy from an alternating magnetic field (AMF) into heat, thus raising the temperature at the side at which MNPs are deposited.^4,6,9,10^ The heat can be used to either induce a direct damage to the tumor or to synergize with other therapies, thus achieving a better treatment outcome.^8^ In order to avoid eddy currents in healthy tissues, the product of field intensity and frequency (*H·f*) has to be lower than 5 × 10^9^ A·m^–1^·s^–1^.^9−12^

In the context of drug delivery, the heat generated from MNPs during MHT has been considered as a promising stimulus to trigger the release of loaded molecules.^4^ Following this approach, drug molecules were linked to the MNP surface via thermal sensitive bonds that could be disrupted at either macroscopic (in the whole media) or local (only vicinal to the MNP surface) temperature changes, the latter also known as the “hot-spot” effect.^6,9,13−16^ Oncotherapy based on MHT treatment at 43–46 °C and at an increased temperature for heat-mediated drug delivery requires a sufficient accumulation of MNPs to ensure that the collective heat dissipation reaches the therapeutic temperature range. Therefore, in these cases, only intratumoral nanoparticle deposition enables to reach a therapeutic dose of MNPs, achieving MHT-mediated temperature increase in the therapeutic range (43–46 °C). Alternatively, considering the local heat effect to release a drug associated to a magnetic nanoplatform is a promising strategy for the systemic administration of the drug, as only a tiny fraction of the injected MNPs homes to tumor, and the drug release will not be triggered by the macroscopic temperature rise, but it will rely on the local MNP heat.^17−21^ Thanks to their thermosensitive feature, Diels–Alder adducts and diazo compounds have been mainly proposed and exploited as linkers to bind the drug molecules to the MNP surface.^19,22−25^ Diazo compounds were widely used as initiators for radical polymerization as they thermally decompose to generate radicals.^26−28^ Interestingly, the decomposing/cleaving temperature can be tuned to values as low as 40 °C by modifying the azo chemical structures.^28,29^ However, handling such compounds is challenging due to their light and temperature sensitivities. In addition, multiple synthetic steps are required to introduce such a linkage between drug molecules and the MNP surface. In contrast, Diels–Alder adducts are employed as chemicals in “click” reactions for materials chemistry and polymer chemistry.^30−33^ This click reaction between diene compounds and dienophile can be carried out at ambient temperature without the need of any catalysts, reaching a quantitative yield. Although the cleaving temperatures of the Diels–Alder adduct (retro-reaction) are rather high (ca. 100 °C) with respect to diazo compounds, a suitable choice of diene and dienophile can decrease the cleaving temperature.^34−37^ Indeed, several studies have shown that the *endo* adduct between furfuryl and maleimide is cleavable at a temperature as low as 60 °C.^36,37^ Moreover, as shown by different groups, during MHT, the temperature at the surface of MNPs was much higher than that of the surrounding media, generating a “hot-spot” effect that induced the breakage of some bonds and triggered the drug release.^8,19^ More specifically, N’guyen and co-workers demonstrated the release of rhodamine (as a drug model) linked via furfuryl–maleimide adducts to the surface of iron oxide nanoparticles by using MHT without the need for a rise of macroscopic temperature.^22^ Despite showing the proof of concept for the AMF-triggered release, the reported magnetic nanocarriers based on diazo or furfuryl–maleimide adducts suffered a low heating performance. Indeed, for these studies, spherical iron oxide MNPs displaying modest SAR values were initially employed as the heat transducers.^18,19,22,25^ As such, the release of the loaded drug was shown only under AMF conditions, out of the clinical and safe range, making the translation of such systems not feasible to the clinic. In addition, the ligand motif used in this study contains only one furfuryl group per ligand molecule, thus enabling the binding of a limited number of dye molecules. This, in turn, results in a low amount of dyes/drugs that can be loaded to the NP surface. Eventually, a sufficient dose of dye molecules was released when using a rather high concentration of Fe (1.75 g·L^–1^).^22,24,25^

Therefore, the development of a high-quality magnetic nanoplatform based on MNPs featuring high SAR values plays a central role for the clinical translation of MHT hot-spot-mediated release systems. Among these, iron oxide nanoparticles of cubic shape, the IONCs employed here, represent one of the promising candidates. Thanks to their magnetic properties strictly related to their unique cubic shape, IONCs exhibits 1 order magnitude higher SAR values with respect to the spherical nanoparticles of the same magnetic volume.^38,39^ The IONCs chosen here were also proved to work as heat transducers for MHT and heat-mediated drug delivery in in vivo models reaching the therapeutic temperature at an iron dose that was 1 order of magnitude lower than that of spherical iron oxide NPs.^14,38−40^ Besides the magnetic properties of the magnetic core, surface ligands have a great impact to ensure the stability of MNPs in physiological conditions and in turn to affect their heating capability which strongly depends on the IONCs colloidal stability and aggregation state.^33,41−47^ Block polymers, owing to their macromolecular structure, can serve as a versatile and robust platform to provide multiple functions of interest.^43^ For instance, the polymer structure can be tailored to bear several surface coordination moieties to anchor the nanoparticle surface, also known as multidentate, which helps to improve the stability of NPs in physiological conditions by reducing the ligand detachment. Modified polymers bearing catechol groups, such as poly(acrylic acid) (PAA) and poly(isobutylene-*co*-maleic anhydride) (PIMA) derivates, have a high affinity for iron oxide-rich surface.^43,48−52^ However, synthetic approaches using these polymers suffer some drawbacks. For PAA, the introduction of catechol and other functional groups, that is, polyethylene glycol (PEG), was done by the reaction of the carboxylic acid group of the polymer toward primary amine-derived molecules using 1-ethyl-3-(3-dimethylaminopropyl)carbodiimide (EDC) coupling chemistry which is not efficient and selective. The ring opening reaction between the anhydride in PIMA and primary amine is more reactive and selective, but it generally results in the formation of carboxylic acid groups on the resulting ligand structure.^43,48,51,52^ Moreover, these commercial polymers are often synthesized by means of free radical polymerization, thus limiting the chances to tune their degree of polymerization, and the polymer chains are not uniform, represented by a high dispersity (Đ) index of 3 to 4. In this respect, using activated ester-based polymer synthesized by living radical polymerization would be more advantageous. Recently, we have shown that the copolymer of N-succinimidyl methacrylate (NSMA) and polyethylene glycol methacrylate represents a robust and versatile precursor to synthesize PEGylated multidentate ligands for water transfer of inorganic nanoparticles.^53^ With the ester NSMA pendants being activated, different anchoring groups can be introduced in a selective and efficient manner. As the polymer precursor already has PEG side chains, the post-synthesis reaction to introduce PEG molecules can be skipped, thus reducing the complexity of the synthetic pathway.

In this study, we report a magnetic nanocarrier based on IONCs (17 ± 2 nm) and a multifunctional polymer that can load and release fluorescein dye, chosen as a drug model, or doxorubicin (Doxo) as a chemotherapeutic agent by means of retro Diels–Alder reaction and under clinically relevant MHT field conditions. Taking advantage of the one-pot reaction between well-defined poly(*N*-succinimidyl methacrylate-*co*-polyethylene glycol methacrylate), P(NSMA-*co*-PEGMA), and dopamine, furfuryl amine, and PEG chains bearing either carboxylic acid (−COOH)- or azide (−N_3_)-terminated groups, we synthesize a versatile and robust polymer ligand (PEG-CF-COOH) bearing several catechol (C) and furfuryl (F) moieties along with functional PEG-COOH pendants. On the other hand, by the reaction of P(NSMA-*co*-PEGMA) with dopamine, furfuryl amine, and amine-PEG chains bearing azide (−N_3_)-terminated groups, a PEG-CF-N_3_ polymer platform was made.

The furfuryl pendants of PEG-CF-COOH reacted with maleimide-derived fluorescein to yield PEG-CFluo-COOH, while PEG-CF-N_3_ was conjugated with maleimide-derived Doxo to yield PEG-CDoxo-N_3_. The surface of IONCs was primed with the developed ligands using a simple and scalable two-step ligand exchange procedure. Here, surfactant-coated IONCs were first treated with tetramethylammonium hydroxide (TMAOH) to transfer the nanocubes into water and temporarily stabilize them by charge repulsion. Next, in the presence of sodium carbonate as a nontoxic base, the addition of the aqueous soluble PEG-CDoxo-N_3_ or PEG-CFluo-COOH to the surface of IONCs enables the ligand exchange of TMAOH molecules with our developed ligands. The SAR values of IONCs upon water transfer are comparable to those values reported in the literature for the same IONCs coated by other polymers. Moreover, they remain colloidally stable in serum solution until 8 days. Most importantly, it was demonstrated here that at clinical MHT conditions as mild as 110 kHz and 16 kA·m^–1^, the release of doxorubicin and fluorescein dye was achieved with a negligible change of macroscopic temperature and with a less dependence on the nanoparticle concentration in solution.

## Results and Discussion

**Figure 1:**
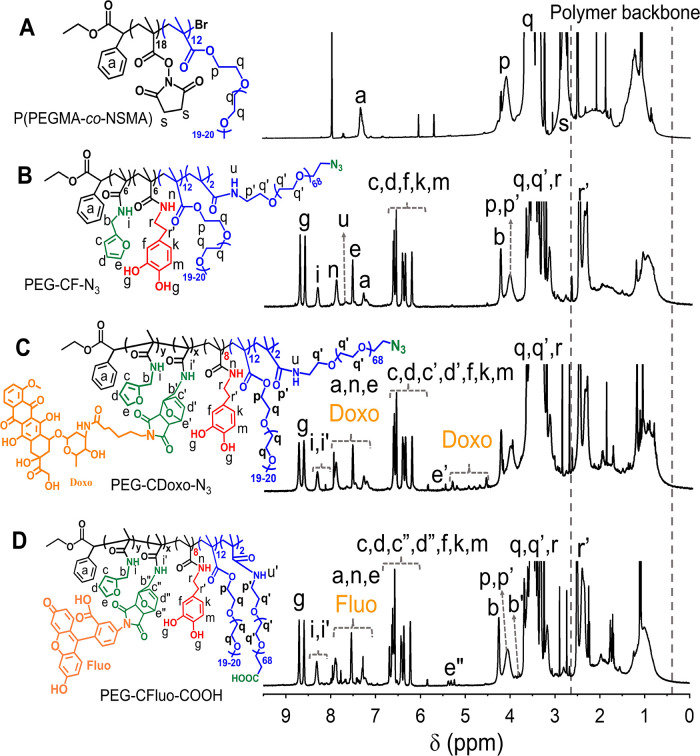
Characterization of multidentate and functional polymers by ^1^H NMR. ^1^H NMR spectra (with the assignments of the characteristic peaks) of P(PEGMA-*co*-NSMA) (A); polymer precursor upon the reaction with NH_2_-PEG-N_3_, furfurylamine, and dopamine hydrochloride (PEG-CF-N_3_) (B); and multifunctional PEGylated polymeric ligand (PEG-CDoxo-N_3_) after the reaction between PEG-CF-N_3_ and maleimide-derived doxorubicin (C); and (D) multifunctional PEGylated polymeric ligand (PEG-CFluo-COOH) after the reaction between PEG-CF-COOH and maleimide-derived fluorescein. The measurements were done using deuterated DMSO as the solvent.

**Scheme 1:**
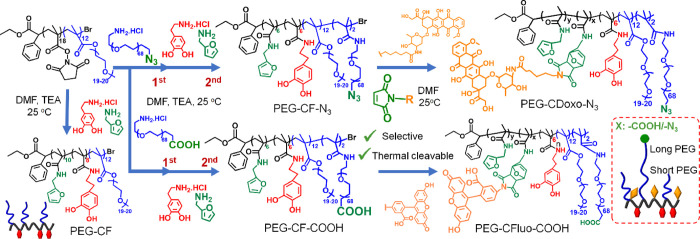
Representative Synthetic Approach To Prepare Multifunctional PEGylated Polymeric Ligands Using Activated Ester Methacrylate-Based Polymers and Diels–Alder Click Chemistry Poly(polyethylene glycol methacrylate-*co*-N-succinimidyl methacrylate), P(PEGMA-*co*-NSMA), is used as a reactive precursor to introduce functional PEG, catechol, and furfuryl pendants by means of a one-pot aminolysis reaction, followed by the conjugation of biomolecules such as maleimide-derived fluorescein (Fluo) or maleimide-derived doxorubicin (Doxo) by Diels–Alder click chemistry.

The synthesis of PEGylated multidentate ligand bearing several furfuryl groups was aimed, taking advantages of photo-induced atom transfer radical polymerization (Photo-ATRP) of activated ester methacrylate, followed by the aminolysis reaction (Scheme 1). Initially, a poly(N-succinimidyl methacrylate-*co*-polyethylene glycol methacrylate), P(NSMA-*co*-PEGMA), was synthesized by the copolymerization reaction of N-succinimidyl methacrylate and polyethylene glycol methacrylate using the Photo-ATRP technique, as described in our previous work (see also Scheme S1 of the Supporting Information).^53^ Here, a molar feeding ratio [NSMA]:[PEGMA]:[Initiator] of 12:8:1 was used. After the reaction time, the polymerization solution was passed through a column of aluminum oxide to remove the copper catalyst, followed by precipitation in diethyl ether to remove the unreacted reagents. The obtained polymer was characterized by ^1^H NMR, and the characteristic proton peaks of succinimidyl (s) and PEG pendants (p,q) can be clearly identified, indicating a successful polymerization step (Figure 1A). By comparing the integration peaks between the phenyl group of the initiator (a) and the methylene group (p) of PEG and the one of succinimidyl (s), a degree of polymerization of 26 and a molar percentage of NSMA of 54% were determined. Size exclusion chromatography (SEC) revealed a molar mass of 13,500 g·mol^–1^ along with a low dispersity (Đ) of 1.25, indicating that the polymerization occurred in a controlled manner.

Next, P(PEGMA-*co*-NSMA) was used as a precursor to synthesize two types of multifunctional polymers, PEG-CF-COOH and PEG-CF-N_3_ as shown in Scheme 1. Here, a longer PEG spacer (*M*_w_ of 3000 g·mol^–1^) was introduced by first carrying out the reaction of P(PEGMA-*co*-NSMA) with NH_2_-PEG-COOH.HCl amino-PEG, followed by the reaction with furfuryl amine (FA) and dopamine (DOPA.HCl) in a one-pot reaction to yield PEG-CF-COOH. The molar ratio between NH_2_-PEG-COOH and NSMA was kept at 1:7, while both FA and DOPA.HCl (anchored via the amino group on the molecules) were used in excess with respect to NSMA (2:1 molar ratio for both). For the production of the PEG-CF-N_3_ polymer ligand, an identical procedure and molar ratios of FA and DOPA.HCl to NSMA were used, except for the replacement of NH_2_-PEG-COOH.HCl with NH_2_-PEG-N_3_, having the same molar mass of 3000 gmol and at the same molar ratio with respect to NSMA. Once these ligands are anchored on the surface of IONCs, the existence of either carboxylic acid or azide groups will offer the possibility to modify with other functional groups such as targeting moieties and/or imaging agents. Both PEG-CF-COOH and PEG-CF-N_3_ were purified by intensive dialysis against 0.01 M HCl solution and final dialysis against MilliQ water prior to the characterization by ^1^H NMR. It was observed that the pH of media used for dialysis had an important role on the purity of the resulting polymer ligands. Indeed, when an aqueous solution at pH 7.4 was used as the medium, the dialysates turned quickly to a reddish color, indicating the self-polymerization of excess dopamine in a basic aqueous solution (data not shown). The reaction seemed to be even further pushed by the addition of TEA, which switched the pH of the media to more basic values. ^1^H NMR performed on the dialysate (after three cycles of dialyzing against water) also revealed the characteristic peaks of poly(dopamine) (8.50 to 7.50 ppm) (Figure S1). Therefore, to avoid the self-polymerization of dopamine, a diluted acidic water solution (HCl 0.01 N) was chosen as the dialyzing medium because at this pH, no change in solution color and no other sign of self-polymerization of dopamine were observed.

Figure 1B depicts the ^1^H NMR spectrum of PEG-CF-N_3_ in which all the characteristic resonances of the polymer can be verified. The retraction of the N-succinimidyl signal (Figure 1A,s) along with the appearance of amide proton (Figure 1B,i) verified the successful reactions between P(PEGMA-*co*-NSMA) and the amino derivatives. By comparing the integration peaks between the primary amide protons i (furfuryl), n (dopamine), u (PEG-N_3_), and the aromatic proton a (phenyl group of the initiator), good agreement was found between the feeding ratio and the actual ones in the polymer, as determined by the integration of the signals of interest in ^1^H NMR (Table S1). A similar observation was made for the case of PEG-CF-COOH as all the characteristic peaks of primary amide protons i′ (furfuryl), n′ (dopamine), u′ (PEG-N3), and the aromatic proton a′ (phenyl group of the initiator) can be visualized (Figure S2), and the ratio between their integration matches well with the designed ones (Table S1). It was determined that each polymer molecule contained six catechol and six furfuryl groups along with two PEG-COOH/-N_3_ chains, while the conversion of NSMA was quantitative. PEG-CF-N_3_ and PEG-CF-COOH polymer ligands bothcontains several furfuryl pendants and hold the capability to conjugate drug molecules bearing the maleimide group. The maleimide conjugation to the furfuryl ligand gives rise to a Diels–Alder adduct that can be cleaved by thermal energy.

To provide the proof of concept of click chemistry functionalization to the furfuryl group, fluorescein-derived maleimide (Fluo-Mal) and doxorubicin-derived maleimide (Doxo-Mal) were selected. Here, Doxo-Mal reacted with PEG-CF-N_3_, and the same chemistry was used to yield PEG-CDoxo-N_3_ by simply mixing PEG-CF-N_3_ with Doxo-Mal, followed by precipitation in diethyl ether. The reaction was carried out in DMF for 6 days at ambient temperature as this temperature is reported to promote the formation of more *endo* adducts than the *exo* one between the maleimido and the furfuryl moieties.^32^ The polymer was purified by several dissolution–precipitation reactions in THF and diethyl ether, respectively. To avoid the risk of compromising the hydrophilicity of the resulting PEG-CDoxo-N_3_, a rather low amount of Doxo-Mal (10% molar equivalent with respect to the amount of furfuryl groups in the feeding polymer in the feeding ratio) was aimed. The ^1^H NMR spectrum of PEG-CDoxo-N_3_ is shown in Figure 1C in which the formation of the Diels–Alder adduct is evident (proton e′ and i′). The integration of i (from 8.50 to 8.25 ppm) and i′ (from 8.25 to 8.00 ppm) revealed a quantitative conjugation of Doxo-Mal to the polymer ligand platform.

On the other hand, Fluo-Mal was conjugated to PEG-CF-COOH to yield PEG-CFluo-COOH. The reaction and purification were done in the same way as shown for PEG-CDoxo-N_3_, except that the ratio of maleimide to furfuryl was fixed to be 25% as Fluo-Mal is less hydrophobic compared to Doxo-Mal. As shown in Figure 1D, all the characteristic peaks of fluorescein and the Diels–Alder adduct were detected in the ^1^H NMR spectrum of PEG-CFluo-COOH, and a quantitative conjugation was also verified. Thanks to its high selectivity, the dye/drug conjugation via the Diels–Alder reaction did not compromise the catechol functionalities as their characteristic peaks (Figure 1D,f,g,k,m) are preserved.

It is worthy to note that we also prepared the ligand polymer without introducing a longer functional PEG spacer to simplify the reaction procedure and make it more straightforward (Scheme S1). In this case, P(PEGMA-*co*-NSMA) was reacted with only a mixture of FA and DOPA.HCl to yield PEG-CF, as shown in the scheme in Figure S2A. The purification step was done in the same way as described above for the other two ligands, and the resulting PEG-CF was subjected to ^1^H NMR. The ^1^H NMR spectrum of PEG-CF shows the peaks of catechol pendants (Figure S3A, f,g,k,m) originated from dopamine pendants, and the characteristic signals of furfuryl moieties (Figure S3A, c, d, e) could also be assigned. In addition, the reduction of N-succinimidyl signal at 2.8 ppm along with the appearance of an amide proton (Figure S3A,n,i) verified successfully the reactions between P(PEGMA-*co*-NSMA) and the amino derivatives. Here, by comparing the integration of aromatic protons with the furfuryl (c, d, e), catechol (f, k, m), and phenyl group linking on the polymer of the initiator (a) (Figure S3A), it was determined that each polymer chain contained six catechol and eight furfuryl groups along with a quantitative conversion of NSMA. The conjugation of Fluo-Mal to PEG-CF to yield PEG-CFluo was also attempted using the same protocol developed for PEG-CFluo-COOH. Here, the ratio between Fluo-Mal to furfuryl was aimed at 66%. The solution after the reaction was washed thoroughly by five cycles of dissolution–precipitation in THF and diethyl ether subsequently. As demonstrated by the ^1^H NMR spectra (Figure S3B), no signal of free Fluo-Mal was detected in the spectrum of PEG-CFluo, while the signal of the Diels–Alder adduct (Figure S3B,e′) was clearly evident. In addition, the ratio between the integration of the Diels–Alder adduct peaks and catechol peaks indicated a quantitative conversion of Fluo-Mal.

**Figure 2:**
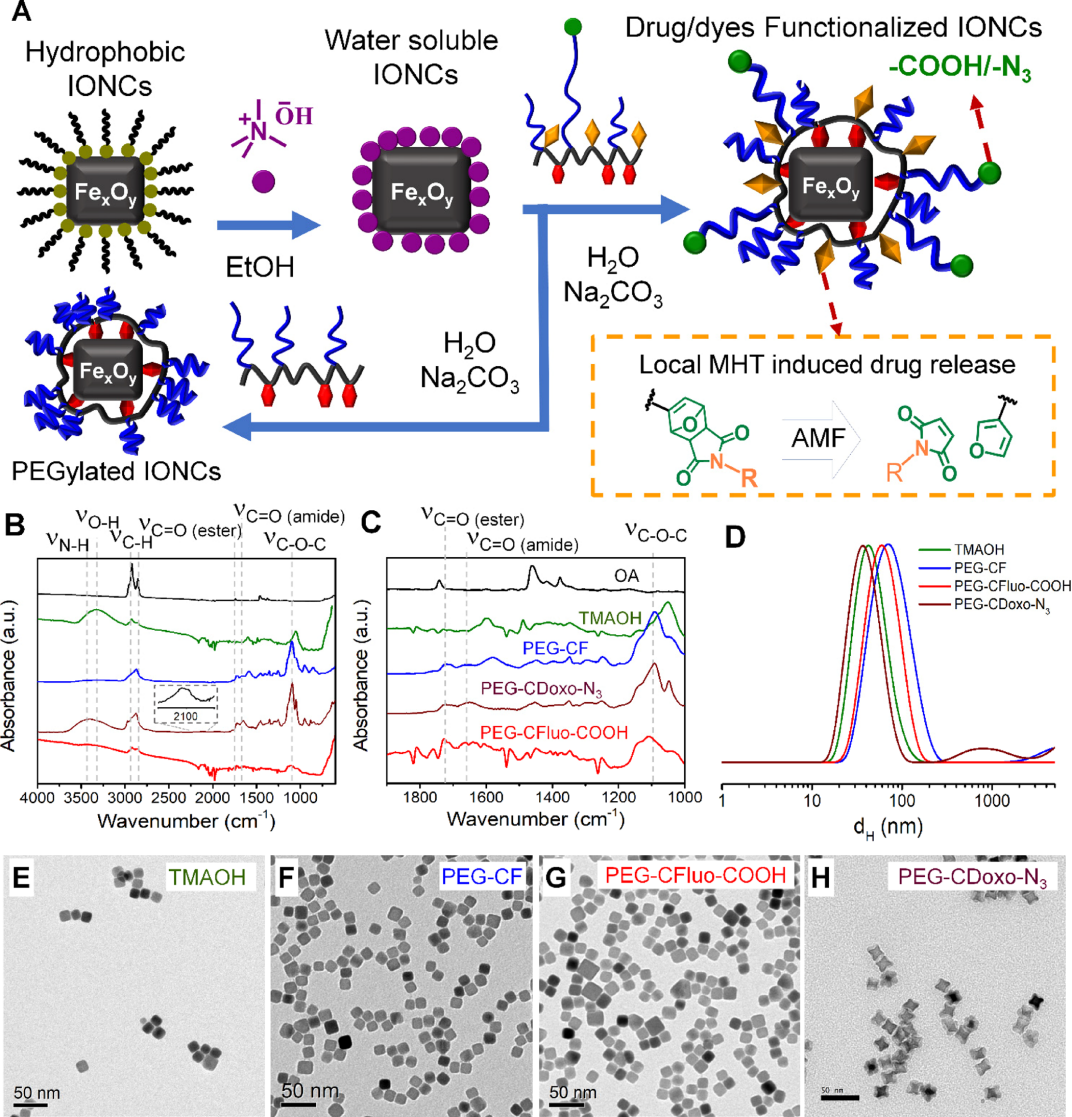
Phase transfer of iron oxide nanocubes (IONCs) using a two-step ligand exchange. (A) Sketch represents the two-step phase transfer procedure involving first the transfer of IONCs from chloroform into water using tetramethylammonium hydroxide (TMAOH), followed by the postexchange in water of TMAOH with the developed ligands in basic solution, to yield physiologically stable IONCs. The dye/drug conjugated to the ligand platform via a thermal labile Diels–Alder adduct could be released by the local heat generated on the nanocube surface during MHT, as illustrated in the inset. FT-IR spectra of surface modification of IONCs for each step of the water transfer protocol (B) and in the extended region of interest from 1000 to 1900 cm^–1^ (C). Dynamic light scattering (DLS) traces of water-soluble IONCs modified with TMAOH (green), PEG-CF (blue), PEG-CFluo-COOH (red), and PEG-CDoxo-N_3_ (deep red) weighted by intensity (D). TEM images of IONCs functionalized with TMAOH (E), or with PEG-CF (F), or with PEG-CFluo-COOH (G), or with PEG-CDoxo-N_3_ (H) deposited from water.

The four synthesized ligands including PEG-CF, PEG-CFluo, PEG-CDoxo-N_3_, and PEG-CFluo-COOH were then used to phase transfer IONCs into water, initially capped with oleic acid, and dispersed in chloroform. For the synthesis of highly monodispersed IONCs, a solvothermal protocol previously reported by some authors was applied.^54^ The chosen IONCs having a cube edge of 17 ± 2 nm were aimed as they have significant SAR values with heating losses that are less susceptible to the viscosity of the surrounding environment, that is, tumor and intracellular compartments, with respect to bigger nanocubes which may heat more, but their heat is significantly affected by viscosity.^55^ In order to obtain single-coated IONCs, we have set a simple and scalable two-step phase transfer protocol, as shown in the scheme in Figure 2A. In the first step, IONCs were transferred from the organic phase into water using tetramethylammonium hydroxide (TMAOH), as reported elsewhere in the literature.^56^ This step is aimed to exploit the electrostatic repulsion of ammonium cations absorbed on the IONCs surface to ensure proper dispersion of the nanocubes in water, the solvent used for the second ligand exchange step (Figure 2A).

The phase transfer of IONCs from chloroform into water was carried out by dispersing IONCs in a TMAOH solution in ethanol which implies a ratio of 175 TMAOH ligands/nm^2^. Sonication at room temperature was used to assist the ligand exchange, followed by the addition of Milli-Q water and centrifugal filtration (see experimental section). This step was repeated twice to wash out the excess of TMAOH ligands. The nanoparticles were finally recovered and redispersed in Milli-Q water. Afterward, an aqueous solution of PEG-CF, used as a ligand model, (100 mg/mL of polymer), was added dropwise to this IONCs–TMAOH solution (12 gFe·L^–1^, 222 μL), followed by the Na_2_CO_3_ base addition (106 mg) to promote the ligand exchange. It is worth to mention that triethylamine (TEA) has been widely used as the base to promote the ligand exchange between catechol-based molecules and the iron oxide-rich surface.^19,48,51^ However, TEA is a hazardous reagent as it is highly volatile with a pungent odor and toxic.^57^ Here, taking advantage of the fact that the ligand exchange can be carried out in water, Na_2_CO_3_ was used as a mild and environment-friendly base to replace TEA. Having a similar pKa value (∼9.0), we expect a comparable performance between TEA and Na_2_CO_3_. Here, upon the addition of Na_2_CO_3_, the nanocubes solution turned milky. A drastic increase in the ionic strength of the solution might induce the flocculation of IONCs in this case.

Keeping the polymer IONCs solution for an overnight reaction under vigorous shaking, led to the phase separation between a black gel containing PEG-CF and IONCs-PEG-CF and Na_2_CO_3_ solution. This black gel was washed several times with water by magnetic decantation. Once the pH of the washed supernatant reached neutrality (ca. 7.0–7.4), this black gel was dispersed in neutral water, and the solution was left under shaking for 24 h until it became clear and homogeneous. The free PEG-CF and traces of Na_2_CO_3_ were removed by centrifugal filtration to yield a clear and stable solution of IONCs-PEG-CF. It is noticed that in a control experiment in which we used TEA instead of Na_2_CO_3_ as the base, it also allowed to obtain a stable and clear solution of IONCs-PEG-CF. In this case, TEA did not cause the formation of the gel, so no magnetic separation was needed, and the IONCs-PEG-CF were simply cleaned by centrifugal filtration after the ligand exchange. As a quick verification of the changes in the IONCs surface functionality, a proof of solubility in water was quickly preformed: by dissolving IONCs-TMAOH or IONCs-PEG-CF- (obtained either when using Na_2_CO_3_ or TEA as bases) in concentrated phosphate-buffered saline (PBS, 0.2 M), while the solution of IONCs–TMAOH in such media turned immediately cloudy and started the phase separation within the first few minutes, leading to sedimentation after 24 h (Figure S4), IONCs-PEG-CF in the same media remained stable without any phase separation even after 24 h. This indicates that the replacement of TMAOH on IONCs with PEG-CF took place and helped to stabilize the nanoparticles. We have also implemented the same procedure to transfer IONCs into water using PEG-CFluo, PEG-CFluo-COOH, and PEG-CDoxo-N_3_. Surprisingly, the IONCs functionalized with PEG-CFluo exhibited low water solubility with the formation of big floating particles even with intensive sonication. Apparently, the conjugation of a high dye amount (dye-to-furfuryl of 66%) makes the polymer ligand more hydrophobic, thus compromising its capability to solubilize IONCs in water. In contrast, PEG-CFluo-COOH and PEG-CDoxo-N_3_ successfully transfer IONCs into water, forming a stable ferrofluidic solution that remained stable during several cycles of centrifugal filtration. The free ligand was removed by means of ultracentrifugation using a sucrose gradient. Interestingly, IONCs-PEG-CFluo-COOH and IONCs-PEG-CDoxo-N_3_ exhibited stability in concentrated PBS buffer comparable to that of IONCs-PEG-CF (Figure S4). The obtained stable IONCs functionalized with different ligands were subjected to further dynamic light scattering (DLS) measurements. It is observed that initially we were using a PEG–CF ligand exchange procedure directly in chloroform (CHCl_3_), as the nanocubes and the polymer were fully soluble in this same solvent according to a previously reported protocol applied for other similar polymers.^53^ In doing so, PEG-CF and IONCs were mixed in CHCl_3_ at a catechol/nm^2^ ratio of 25 along with an excess amount of triethylamine (500 TEA/nm^2^) and left to react for an overnight period. Afterward, an excess amount of hexane was added to induce the precipitation of particles. Upon centrifugation, the supernatant was discarded, and the resulting black pellet was dried under nitrogen flow, followed by water addition. The free ligand was removed by centrifugal filtration. Although the IONCs upon ligand exchange can be readily dispersed in water, TEM revealed a predominant presence of small clusters of IONCs together with individually coated nanocubes (Figure S5A,B). This observation is in good agreement with DLS measurements in which the IONCs showed a high hydrodynamic size (*d*_H_) of ∼200 nm weighted by intensity (Figure S5C). This might be due to a strong magnetic interaction between IONCs as they have the magnetic core size and distribution at the edge between superparamagnetic and ferromagnetic.^14,38^ As reported in our previous studies, such clustering may compromise the MHT heating capability of IONCs either in solution or in viscous media;^41^ moreover, having a large *d*_H_ implies that these clusters are likely to be less desirable for systemic delivery. We therefore chose the two-step approach with the intermediate solubilization in water via TMAOH for a better final water-soluble product.

The evolution of IONCs’ surface functionality was further confirmed by the FT-IR fingerprint of the same nanocube at each of the functionalization steps. The FT-IR spectra of oleic acid-capped pristine IONCs show the absorption bands of the stretching vibrations of alkyl (3000–2800 cm^–1^), carbonyl (1710 cm^–1^), and carboxylate (1490 cm^–1^) groups, indicating the presence of oleic acid as the surface ligand (Figure 2B,C, black curve). Upon the phase transfer using TMAOH, the characteristic absorption bands of hydroxyl (−OH) and C–N at 3300 and 1600 cm^–1^, respectively, can be clearly identified (green curve). The FT-IR spectra of IONCs-PEG-CF (blue curve) show the appearance of distinguished signals of carbonyl stretching at 1720 cm^–1^ (ester) and 1652 cm^–1^ (amide), along with the strong and characteristic bands of the ether bond (C–O–C) at 1100 cm^–1^ which confirm the existence of PEG-CF on the surface of IONCs. A similar observation was made for IONCs-PEG-CFluo-COOH and PEG-CDoxo-N_3_. Here, FT-IR measurements (Figure 2B,C) confirmed the existence of PEG-CFluo-COOH (red curve) and PEG-CDoxo-N_3_ (deep red curve) on the surface of IONCs upon the modification step, as the characteristic absorption bands of carbonyl (C=O, ester, and amide) and ether (C–O–C) can be clearly assigned to 1722, 1656, and 1105 cm^–1^, respectively. In the case of IONCs-PEG-CDoxo-N_3_ (Figure 2B, inset), a characteristic absorption band of azide group at 2150 cm^–1^ can also be detected, confirming the presence of PEG-CDoxo-N_3_ on the surface of IONCs.

The colloidal properties of IONCs during the surface modification steps were monitored by means of DLS. *d*_H_ weighted by intensity, volume, and number along with the polydispersity index (PDI) for each sample is reported in Table 1. As shown in Figure 2D, the DLS traces of IONCs transferred in water using TMAOH showed a single peak along with a small hydrodynamic diameter (*d*_H_) of 48 ± 20 nm (the maximum peak value weighted by intensity with the half width at half-maximum) and a low PDI of 0.12 with no sign of aggregation (black curve). Even the *d*_H_ weighted by volume (35 ± 14 nm) and number (27 ± 8 nm) further verified that TMAOH-IONCs were present as a single entity in water solution (Figure S6A,B). Interestingly, the replacement of TMAOH with PEG-CF on the IONCs’ surface (using Na_2_CO_3_ as the base) led to an increase of their *d*_H_ to 87 ± 42 nm (weighted by intensity) without any traces of aggregation, while the PDI remains as low as 0.20 (Figure 2D, red curve). The corresponding *d*_H_ values weighted by number and volume are also consistent with the one weighted by intensity (Table 1 and Figure S6A,B). On the other hand, *d*_H_ (weighted by intensity) of IONCs-PEG-CDoxo-N_3_ showed the main peak at 41 ± 16 nm (PDI ∼ 0.29), indicating an individual coating of IONCs in solution (Figures 2D and S6A,B) for the DLS traces weighted by volume and number. For IONCs-PEG-CFluo-COOH, the DLS data revealed a *d*_H_ value of 76 ± 33 nm (weighted by intensity) along with a PDI of 0.29, also supported by the same monomodal traces of *d*_H_ values weighted by number and volume (Figures 2D, S6 and Table 1), thus verifying the good colloidal stability. Interestingly, IONCs capped with PEG-CFluo-COOH also exhibit good stability in PBS (50 mM, pH 7.4), as demonstrated by DLS measurements. As shown in Figure S7A, *d*_H_ (weighted by intensity) of PEG-CFluo-COOH in PBS was found to be as small as 61 ± 36 nm (PDI 0.18) and remained unchanged after 21 days of storage on bench under ambient conditions (Figure S7B) as well as the DLS traces weighted by number and volume (Figure S7). It is observed that even for the IONCs-CF-N_3_ and IONCs-PEG-CDoxo-N_3_ samples, the nanocubes before and after Doxo functionalization were both stable in PBS, as confirmed by the monomodal DLS traces (Figure S7C). The data from DLS are in good agreement with those of TEM. As can be seen in Figure 2E–H, IONCs functionalized with TMAOH (deposited from water) are well dispersed on the TEM grid with a few grouping of nanocubes likely due to the drying effect (Figure 2E). The same observation was made in the case of IONCs-PEG-CF, IONCs-PEG-CFluo-COOH, and IONCs-PEG-CDoxo-N_3_ as IONCs form a monolayer of nanoparticles and no apparent signs of aggregation were detected (Figure 2F–H).

**Table 1:** Hydrodynamic Size (*d*_H_) of IONCs Modified with TMAOH, PEG-CF, PEG-CFluo-COOH, and PEG-CDoxo-N_3_ Weighted by Intensity, Volume, and Number along with PDI Values

sample	*d*_H_ intensity-weighted (nm)	*d*_H_ volume-weighted (nm)	*d*_H_ number-weighted (nm)	PDI
IONCs-TMAOH	48 ± 20	35 ± 14	27 ± 8	0.12
IONCs-PEG-CF	87 ± 42	47 ± 28	28 ± 11	0.20
IONCs-PEG-CFluo-COOH	76 ± 33	48 ± 23	34 ± 11	0.29
IONCs-PEG-CDoxo-N_3_	41 ± 16	28 ± 10	22 ± 6	0.29

**Figure 3:**
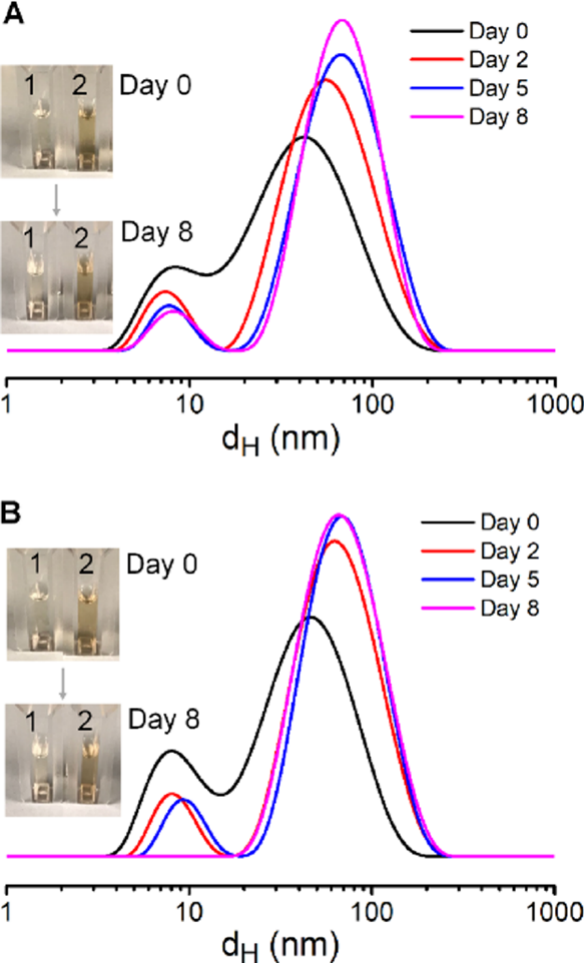
Stability of IONCs functionalized with multidentate and functional polymer ligands in physiological conditions. DLS traces of IONCs modified with PEG-CDoxo-N_3_ (A) or with PEG-CF-N_3_ (B) dispersed in complete cell culture media at 10% fetal bovine serum at day 0 and after 2, 5, and 8 days of storage at ambient conditions. The insets show the vials, as observed under visible light for the culture media (1) and for IONCs modified with either PEG-CDoxo-N_3_ or PEG-CF-N_3_ ligands, respectively (2).

Aiming at applying this platform to tumor cells, the stability of IONCs functionalized with PEG-CDoxo-N_3_ was investigated. In this experiment, surface-modified IONCs were dissolved in DMEM tumor cell culture media at 10% fetal bovine serum (FBS) at [Fe] ∼ 0.1 g/L. The stability of IONCs-PEG-CDoxo-N_3_ in such a solution was monitored at room temperature by visual inspection and DLS characterization at days 0, 2, 5, and 8 days (Figure 3A). The DLS traces (weighted by intensity) of IONCs-PEG-CF-N_3_ at day 0 revealed a bimodal size distribution. The small peak at 9 ± 3 nm might be due to the existence of serum protein in solution, while the peak at 51 ± 27 nm is compatible with our IONCs. The increase in IONCs-PEG-CDoxo-N_3_ could be attributed to the formation of protein corona on the NP surface. At 8 days of incubation, the *d*_H_ value of IONCs-PEG-CDoxo-N_3_ increased gradually up to 77 ± 35 nm. This could be explained by the increase of protein corona or slow clustering of IONCs. However, a *d*_H_ value below 100 nm is still suitable for efficient accumulation at the solid tumor. Notably, even after 8 days, no signs of sedimentation are observed, and the IONC solution still appeared homogeneous (Figure 3A, inset). We also performed the same stability experiment for IONCs functionalized with PEG-CF-N_3_. Also in this case, similar observations were made as the *d*_H_ value of IONCs-PEG-CF-N_3_ in 10% FBS remained below 100 nm (Figure 3B), and no visual precipitates (Figure 3B, inset) were detected in solution up to 8 days of incubation. As such, the conjugation of Doxo-Mal resulted in a negligible impact on the capability of PEG-CF-N_3_ to stabilize IONCs in media.

**Figure 4:**
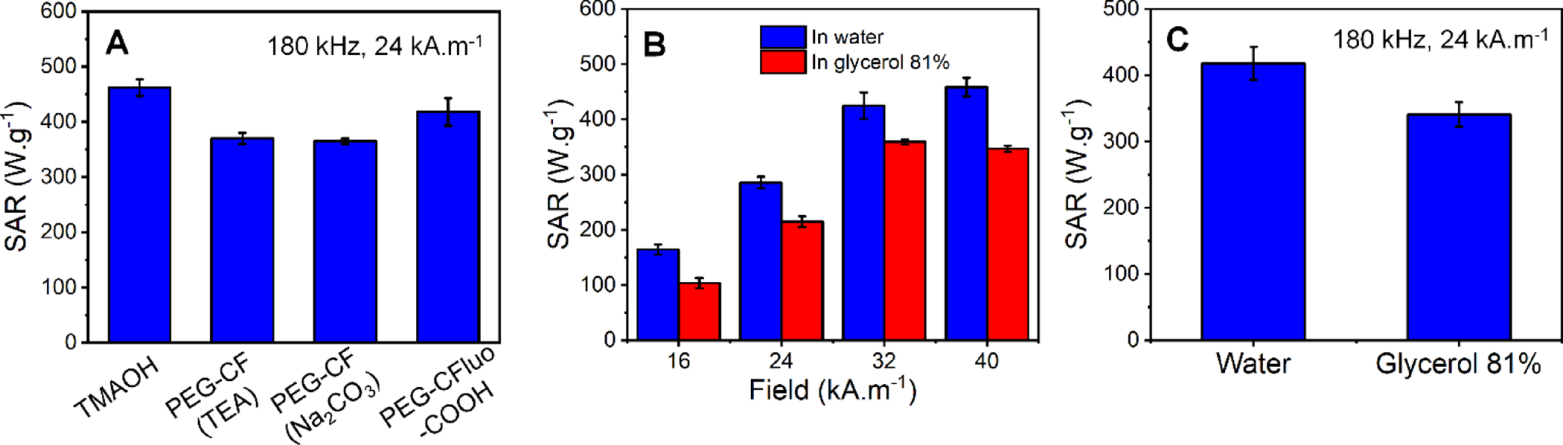
Heating capability of IONCs in water and viscous media. (A) Specific absorption rate (SAR) values in water of IONCs having different surface ligands. (B,C) Comparison of the SAR value of IONCs functionalized with PEG-CFluo-COOH in water and viscous media (glycerol 81%) measured under the MHT conditions with respect to the biological limit (*H*·*f* < 5 × 10^9^ A·m^–1^·s^–1^). The values reported in panel 4B were measured at 110 kHz, with the field varied from 16 to 40 kA·m^–1^.

Thanks to the high colloidal stability, IONCs capped with TMAOH and PEG-CF showed SAR values above 350 W·g^–1^, measured using the calorimetric method under MHT conditions (24 kA·m^–1^ and 180 kHz) and below the biological limit (*H*·*f* < 5 × 10^9^ A·m^–1^·s^–1^). Here, IONCs–TMAOH shows an outstanding SAR value of 460 W·g^–1^ (Figure 4A), while changing the surface ligand to PEG-CF led to a slight drop of SAR by 20%, regardless of the use of organic (TEA) or inorganic base (Na_2_CO_3_). Owing to its good stability, IONC-PEG-CFluo-COOH also shows a SAR value as high as 420 W·g^–1^ (Figure 4A) when being exposed to an MHT with respect to the biological limit (24 kA·m^–1^, 180 kHz). This SAR value was found to be slightly lower than that of IONC–TMAOH (drop by 10%) but higher than the value measured for IONC-PEG-CF (360 W·g^–1^). We next investigated the SAR value of IONC-PEG-CFluo-COOH in viscous media (Figure 4B). Indeed, as the viscosity in tumor and intracellular compartments is far higher than that in water, the evaluation of the heating capability in viscous media is an important criterion to realize the actual potential of the heat transducer in MHT.^41,55,58^ The SAR measurements on IONC-PEG-CFluo-COOH were therefore performed in water and in glycerol solution (81%) at the same Fe concentration (3.5 g/L). Here, a clinically used frequency of 110 kHz was aimed while the field amplitude was varied from 16 to 40 kA·m^–1^ to ensure that the *H·f* factor was always below 5 × 10^9^ A·m^–1^·s^–1^. Notably, we found that SAR of IONC-PEG-CFluo-COOH in water increased linearly as a function of field amplitude in the range from 16 to 32 kA·m^–1^, while there was no significant difference between the SAR values measured at 32 and 40 kA·m^–1^ (Figure 4B). A similar trend was obtained for the same IONC-PEG-CFluo-COOH dispersed in 81% glycerol solution, and the SAR value of IONC-PEG-CFluo-COOH in glycerol solution did not decrease when the viscosity of the media was increased from 1.0 to 91.0 mPa·s (Figure 4B). Here, drops by 15–35% were observed depending on the field of the measurement. Apparently, the intermediate field amplitudes (24 and 32 kA·m^–1^) resulted in a less dependence of heating capacity of IONCs-PEG-CFluo-COOH on the media viscosity. This could be due to some alignments of IONCs in high viscous media in these field conditions that enhance their heating performance.^59^ SAR values measured under other MHT conditions (24 kA·m^–1^ and 180 kHz) in viscous media also showed only a marginal drop by 18% (Figure 4C). It is worthy to mention that IONCs functionalized with PEG-CFluo-COOH in our study have among the highest SAR value reported for iron oxide-based nanoparticles (having a similar lateral size for nanocubes or magnetic volume for spherical nanoparticles) in such highly viscous media.^41,58,60^ Although detailed investigations on SAR of IONCs-PEG-CDoxo-N_3_ were not conducted in our current study, we believe that the heating performance of such IONCs is comparable to IONC-PEG-CFluo-COOH as these two samples have the same edge sizes and exhibit a similar colloidal stability, represented by comparable *d*_H_. As such, IONCs functionalized with either PEG-CFluo-COOH or PEG-CDoxo-N_3_ represent promising candidates for practical MHT against solid tumor (Figure 4).

**Figure 5:**
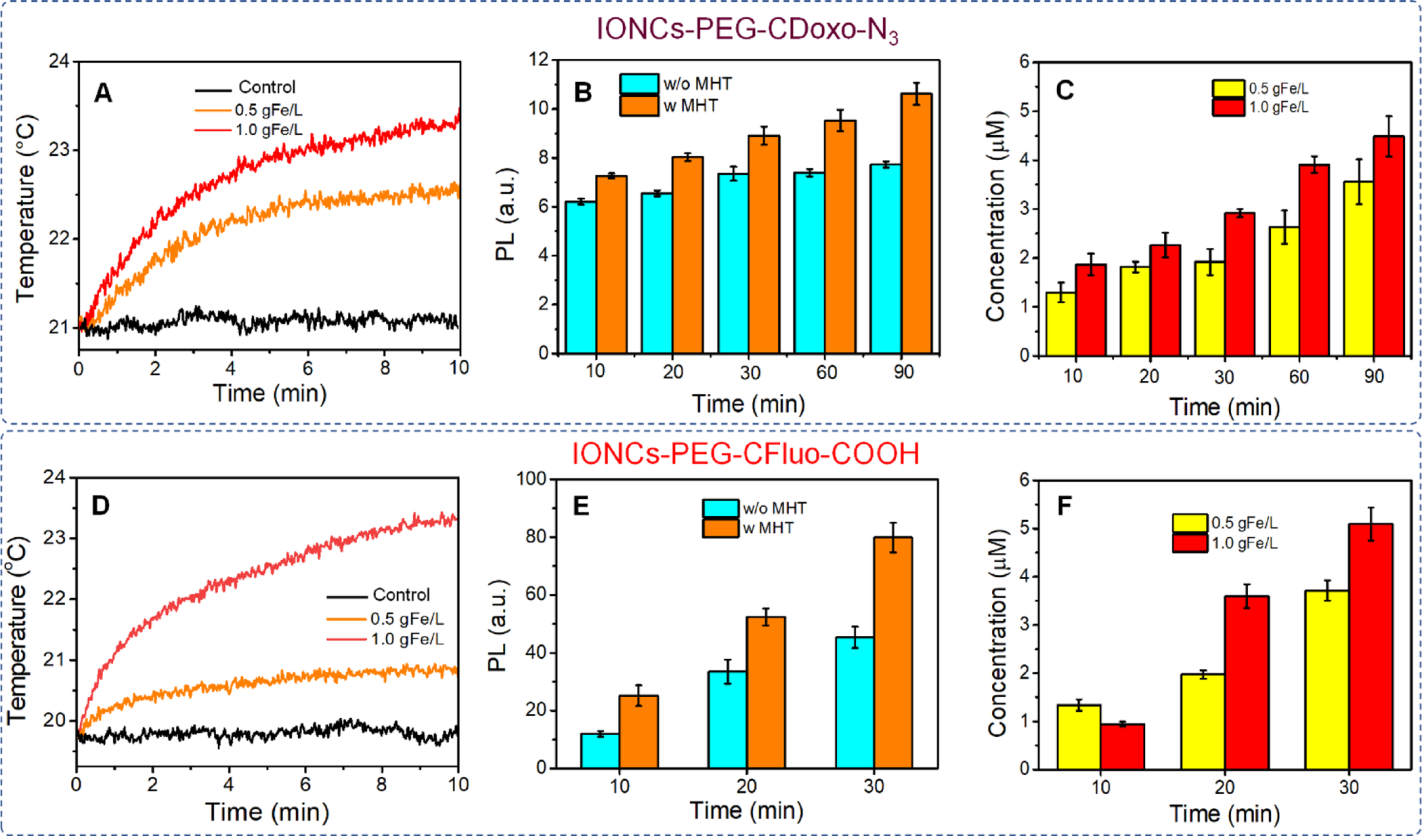
Release of dye molecules by means of MHT-induced local (hot-spot) heat effect. Heating profiles of IONC-PEG-CDoxo-N_3_ (A) and IONC-PEG-CFluo-COOH (D) solution in water at different Fe concentrations (0.5 and 1.0 g/L) and control solution (only water) under MHT (16 kA·m^–1^ and 110 kHz) during the first 10 min of MHT. We observed that the maximum temperature was reached after 10 min; thus, further heating profiles are not shown. The comparison of the PL signal of Doxo (B) and fluorescein sodium salt (E) between the samples kept on bench and the one undergoing MHT (110 kHz, 16 kA·m^–1^) at the Fe concentration of 0.5 gFe/L (at different durations of MHT). The normalized concentration of Doxo (C) and fluorescein sodium salt (F) released upon MHT (16 kA·m^–1^ and 110 kHz) at Fe concentrations of 0.5 gFe/L.

We next used IONCs-PEG-CDoxo-N_3_ and IONCs-PEG-CFluo-COOH as models to study the release of active molecules linked to IONCs via the thermal labile Diels–Alder adduct. The temperature at the surface of IONCs during MHT is far higher than the one in solution;^18,19^ thus, the ability to inducing the retro-Diels-Alder reaction (the breakage) to achieve the release without the need of heating the whole solution was aimed. In the first set of experiments, IONCs-PEG-CDoxo-N_3_ was used. Given the very low solubility of Doxo-Mal in water, this experiment was carried out in H_2_O/DMF (50% in volume) as the release media. To attest the percentage of Doxo loaded, the IONCs-PEG-CDoxo-N_3_ solution was immersed in a water bath set at 70 °C. At this temperature, the cleavage of the *endo* adduct occurs, being this temperature above 60 °C. The supernatant, after the treatment, was separated by means of centrifugation at a high speed, followed by one cycle of magnetic decantation. An attempt to use centrifugal filtration to separate the supernatant was not successful as Doxo was found to be stuck to the filtering membrane (data not shown). The final supernatant was subjected to photoluminescence (PL) measurement. Here, we noticed that the amount of PL signal of the supernatant remained the same between 4 and 7 h of water bath treatment, indicating a complete thermal cleaving of the *endo* adduct. After subtracting the PL signal from the non-specific release, a loading capacity of 46 μg Doxo per 1 mgFe was determined using the calibration of Doxo–Mal in the same media (Figure S8). It is important to note that the actual loaded amount of Doxo will be higher; however, we only considered the one that can be cleaved via mild thermal treatment in this loading capacity determination. We next investigated the release carried out at [Fe] as low as 0.5 g/L with and without MHT exposure. Here, we used an identical MHT condition that has been used in clinic (16 kA·m^–1^ and 110 kHz). The sample was exposed to such MHT condition for different periods (from 10 to 90 min) As shown in Figure 5A (orange curve), the temperature rise in this MHT condition was negligible with an increment of less than 1.0 °C (solution temperature reached 21 °C, and the temperature remained unchanged in the control experiment without IONCs). The amount of released Doxo was measured by the photoluminescence (PL) intensity of collected supernatants (excitation of 475 nm and emission of 593 nm). The PL of the sample exposed to MHT is evidently higher than that of the one kept at room conditions (Figure 5B). The non-specific release in the case of samples kept at room temperature and not exposed to MHT might be due to a small amount of leaching ligands during the centrifugation step used to separate the free Doxo from the nanocube fraction.^19,22^ Similar observations were made when comparing the release at different time periods. The PL intensity of the sample exposed to 90 min of MHT is 50% higher than that of the non-treated one (Figure 5C). The same trend of release was obtained when a solution having higher Fe concentration was used (1.0 g/L) (Figure S9A). In this case, the solution temperature increased by 3 °C, reaching 23 °C (Figure 5A, red curve) during the MHT treatment. Here, 11.3 and 7.1% of loaded Doxo were released after 90 min of MHT at [Fe] of 0.5 and 1.0 g/L, respectively. More importantly, we found that the normalized concentration of Doxo released (calculated by excluding the PL contribution from the nonspecific release) even at low Fe concentration used (0.5 g/L) after 10–90 min of MHT is in the range from 1.3 to 3.6 μM (Figure 5C). The concentration of released drug was determined using a calibration curve of Doxo–Mal in H_2_O:DMF (50% volume) (Figure S8). When a solution having higher Fe concentration was used, the concentration of Doxo released falls in the range between 1.9 and 4.5 μM (Figure 5C).

Next, the release experiment was performed on IONC-PEG-CFuo-COOH. Compared to the experiment with IONC-CDoxo-N_3_, a similar increase of temperature was obtained in this case during the MHT at [Fe] of 0.5 and 1.0 g/L (Figure 5D). Thanks to the better solubility of the cleaved fluorescein sodium salt, the supernatant after MHT could be separated by centrifugal filtration as the released dyes could pass through the filtering membrane easily. As expected, the amount of dyes released for the sample undergoing MHT at 10, 20, and 30 min is always higher than the one left on bench. After 30 min of MHT, the photoluminescence signals of MHT-treated samples are almost double in comparison to the one of the nontreated one for [Fe] of 0.5 and 1.0 g/L (Figures 5E and S9B). Interestingly, we found that the signal from the nonspecific release was much less with respect to the case of IONC-PEG-CDoxo-N_3_. Such difference might be due to the difference in the centrifugal speed used to precipitate out the nanocubes from the solution. Indeed, due to the very high colloidal stability of IONC-PEG-CDoxo-N3 in water, a very high spinning speed (14,500 rpm) was needed to sediment the nanocubes, leading to a more shear-thinning force. This, in turn, will induce more ligand leaching from the IONC surface. Nevertheless, the normalized concentration of dyes released at [Fe] of 0.5 g/L after 10 to 30 min of MHT is in the range from 1.0 to 4.0 μM (Figure 5F) using a calibration curve of fluorescein sodium salt in water (Figure S10). When a solution having a higher dose of nanocubes was used (1.0 g/L in iron), higher amounts of dyes were released in the range between 0.9 and 5.1 μM (Figure 5F).

Notably, these μM concentration ranges in the case of either Doxo or fluorescein release are in the therapeutic window of most antitumor chemotherapies that have been used to treat cancer.^22,24,25,61^ As such, our developed drug ligand IONC platform may enable the release of sufficient amount of drug by means of magnetic induction for clinical use and at a nanoparticle dose quite low as never achieved before.

## Conclusions

We have disclosed a novel polydentate and multifunctional polymer ligand for the preparation of highly stable iron oxide nanocubes (IONCs) in physiological conditions. We took advantage of the high reactivity of poly(polyethylene glycol methacrylate-*co*-*N*-succinimidyl methacrylate) toward primary amines to prepare well-defined polymer ligands having several azide- and carboxylic acid-functionalized polyethylene glycol chains, catechol, and furfuryl pendants. Multiple catechol anchors provide a strong binding of this ligand platform to the iron oxide nanocube surface, while functional PEG chains ensure an excellent stability of IONCs in buffer saline solution or enable to control the hydrophilicity of the polymer ligand molecule, along with the possibility to post-functionalize with the desired biomolecules. Indeed, the furfuryl side groups enable the conjugation of molecule of interest (here, a dye molecule and doxorubicin as drug model systems) via a thermal labile Diels–Alder adduct. A two-step post-ligand exchange procedure was used for water transfer of IONCs. IONCs transferred in water using the developed ligand platform show outstanding SAR values as high as 420 W·g^–1^ when being exposed to an alternating magnetic field of biological limit. It is remarkable that SARs of IONCs capped with a polymer ligand dropped by only 15–30% when the measurements were performed on the sample dispersed in viscous media (91 mPa·s). Most importantly, the release of conjugated dyes/drugs can be triggered by exposing IONCs to MHT condition applied in the clinic (16 kA·m^–1^, 110 kHz) when measuring a change in temperature of only few degrees. Peculiarly, at a nanocube concentration as low as 0.5 or 1 g/L of iron corresponding to the dose of nanocubes of as low as 0.05 or 0.1 mg (considering a tumor volume of 100 mm^3^ in the in vivo model), the amount of released dyes/drugs from nanocubes under MHT was in the dose range 1.0–5.0 μM, being in line with the therapeutic range of various chemotherapies used for cancer treatment. This platform is especially appealing in the case of systemic injection of MNPs when the accumulation dose at the tumor often is not enough to guarantee the macroscopic temperature elevation for drug release. The AMF in clinical conditions could be used, indeed, as the remote actuator for the drug release activation. In addition, the versatility of this established polymer ligand platform can be further exploited to link different functional molecules via Diels–Alder click chemistry, thus enabling the exploration of the new applications of IONCs for mild MHT-activated anticancer therapies.

## Supporting Information Available

Description of materials, synthesis, ^1^H NMR of polymer ligand, TEM images and DLS traces of IONCs, and release profile of dye/drug with and without MHT activation (PDF)
