# Supplementary material for: Clickable Polymer Ligand-Functionalized Iron Oxide Nanocubes: A Promising Nanoplatform for ‘Local Hot Spots’ Magnetically Triggered Drug Release
Source: ACS Appl Mater Interfaces. 2022 Oct 18;14(43):48476–88. doi: 10.1021/acsami.2c14752 (PMC9634696; doi:10.1021/acsami.2c14752)
Supplement: Supplementary file 1 — am2c14752_si_001.pdf [file am2c14752_si_001.pdf]

Supporting Information

Clickable Polymer Ligand Functionalized Iron Oxide Nanocubes: A Promising Nanoplatform for 'local hot spots' Magnetically Triggered Drug Release

Binh T. Mai*[1], John S. Conteh[1], Helena Gavilán[1], Alessandro Di Girolamo[1] and Teresa Pellegrino[1]*

Istituto Italiano di Tecnologia, via Morego 30, 16163, Genova

*Corresponding authors: Teresa.Pellegrino@iit.it ; maithanhbinh1988@gmail.com

Dr. Binh T. Mai

Present address: School of Pharmacy and Pharmaceutical Sciences, Trinity College Dublin, Dublin, Ireland

Dr. H. Gavilán

Present address: Departamento de Ciencia e Ingeniería de Materiales e Ingeniería Química, IAAB, Universidad Carlos III de Madrid, Avda. de la Universidad, 30, 28911 Leganés, Madrid, Spain

Materials

Triethylamine (TEA, Merck, 99%), PEGMA (Mw = 950 g·mol^{-1}) (Merck, 95%), Furfuryl Amine (FA, Merck, ≥ 99%), ethyl phenyl bromoacetate (Merck, 97%), N,N,N′,N″,N″-pentamethyldiethylenetriamine (PMDETA, Merck, 99%), $CuBr_2$ (99%, Merck), Fluorescein Maleimide (Fluo-Mal, ThermoFisher), tetramethylammonium hydroxide (TMAOH, 99%, Merck), Na_2CO_3 (99%, Merck), glycerol (99%, Merck), aluminum oxide (activated, basic, Brockmann I, Merck) and NH_2-PEG-COOH·HCl (Mw = 3000 g·mol^{-1}, RappPolymere), were purchased and used as received. N-succinimidyl methacrylate (NSMA) was synthesized by the esterification between N-hydroxyl succinimide (NHS) and methacryloyl chloride as described in our previous study.[1] All the solvents were purchased from commercial sources with the highest available purity, and they were used as received.

Synthesis of maleimide-derived doxorubicin

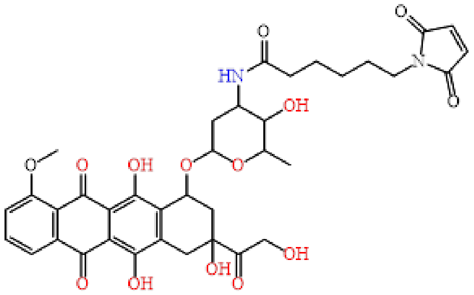

The synthesis of maleimide-derived doxorubicin was carried out following a procedure reported in literature.[2] To an 8 mL vial containing 3.0 mL DMF, Doxorubicin hydrochloride (50 mg, 0.086 mmol, 1 equiv), 6-maleimidocaproylic acid N-hydroxysuccinimide ester (29.2 mg, 0.095 mmol, 1.1 equiv), and Triethyl amine (23.9 µL, 0.174 mmol, 2 equivalent) were dissolved and stirred for 2 h at room temperature. To the solution diethyl ether (4 mL) was added to precipitate the product and filtered through a Sartorius filter paper (292a) to remove the NHS by-product. The reddish filtrate solution was evaporated with rotavapor (0 mbar, bath temperature 55°C) to remove DMF before completely dried in Vacuum (schlenk line) to obtain red crystals (yield 53.6 mg, 0.072 mmol; ca. 85%). The structure of the Doxo-Mal product was confirmed by ^{1}H NMR spectroscopy. 1H NMR (DMSO-d_6, 400 MHz): δ 7.95 (s, 1H), 7.91 (d, 2H), 7.65 (m, 1H), 7.49 (d, 1H), 6.95 (s, 2H), 5.49 (s, 2H), 5.21 (d, 1H), 4.92 (t,1H), 4.89 (t, 1H), 4.75 (d, 2H), 4.59 (d, 1H), 4.15 (q, 1H), 3.99 (s, 2H), 2.98 (t, 1H).

Characterization

The NMR spectra were measured using a Bruker 400 MHz BBI spectrometer with deuterated DMSO as a solvent at 25 °C. Size-exclusion chromatography (SEC) measurements were performed using an Agilent 1260 Infinity quaternary LC system consisting of an Agilent 1260 Infinity quaternary pump (G1311B), autosampler (G1329B), two PLGel 5μm MIXED-C columns (kept at 25 °C) and a refractive index detector (G1362A). THF (with BHT as inhibitor) was used as an eluent at a flow rate of 1 mL/min. The molar masses were determined using Agilent narrow molecular mass distribution polystyrene standards in THF. The particle sizes were characterized by dynamic light scattering (DLS) using a Malvern Instruments Zetasizer Nano series instrument. An equilibration time of 120 seconds was allowed prior to each reading, and measurements were done in triplicate.

Transmission Electron Microscopy (TEM) images were recorded using a JEOL JEM 1011 electron microscope, equipped with a W thermionic electron source and a 11Mp Orius CCD Camera (Gatan company, USA), with an acceleration voltage of 100 kV. The samples were prepared by placing a drop of the sample onto a carbon coated copper grid, which was then left to be dried at ambient condition before being subjected to the microscope.

An elemental analysis was carried out *via* Inductively Coupled Plasma (ICP) Optical Emission Spectroscopy on a ThermoFisher CAP 6000 series. The samples were prepared by digesting 10 µL of the sample in 1.0 mL of aqua regia overnight, followed by dilution with Milli-Q water to 10 mL.

PhotoLuminescent (PL) spectra were measured using a Cary500 eclipse spectrometer, and the excitation wavelength was fixed at 480 nm. To determine the concentration of released dyes after MHT, a calibration curve was constructed using fluorescein sodium salt solution in water with different dye concentration ranging from 0.75 to 10.5 μM. For doxorubicin loading and release experiment, solutions of Doxo-Mal in H_2O/DMF (50% volume) having concentration from 1.0 to 40.0 (µg/mL) was used for the calibration curve.

FT-IR spectra were measured on a Bruker vertex 70v Fourier transform infrared spectrometer.

SAR measurements were carried out by a commercially available DM100 Series (NanoScale Biomagnetics Corp.) device. 150 µL of the sample ([Fe] = 3.0-4.0 $g \cdot L^{-1}$) was added to a cornical vial and exposed to an AC magnetic field at different field amplitudes and frequencies. The

measurement for each samples were performed in triplicate. SAR values were calculated according to the equation

$$SAR\ (W.g^{-1}) \ = \frac{C}{m} \times \frac{dT}{dt}$$

in which C is the specific heat capacity of the solvent (C_{water} = 4185 J·L^{-1}·K^{-1}, $C_{glycerol\ 81\%}$ = 2660J·L^{-1}·K^{-1})[3-4] and m is the concentration (g·L^{-1} of Fe) of magnetic material in solution. As the measurements were carried out under non-adiabatic conditions, the slope of the curve $\frac{dT}{dt}$ was measured by taking into account only the first few seconds of the curve.

Synthesis of polymer having activated ester and PEG pendant (P(NSMA-*co*-PEGMA))

The copolymerization of PEGMA (M_w = 950 g·mol^{-1}) and NSMA was carried out by taking advantage of the Photo-ATRP process. In a typical procedure, PEGMA (Mw = 950 g·mol^{-1}) (7.6 g) was weighed in a 40 mL glass vial, and 8 mL DMF solvent was added. The monomer was dissolved by means of magnetic stirring for 30 min. After that, NSMA (2.18 g) was added and the solution was stirred for further 15 minutes to completely dissolve the monomers. Subsequently, ethyl phenyl bromoacetate (175 µL) and PMDETA (26.9 µL) were added to the solution. The vial was sealed with a cap with septum and purged with a nitrogen flow for 10 min. In the meantime, a stock solution of $CuBr_2$ in DMF (3.2 mg·mL^{-1}) was prepared and also purged with a nitrogen flow for 10 min. After, 3.0 mL of a copper catalyst solution was injected in one shot into the glass vial containing the monomers, ligand and initiator. The resulting solution was stirred for a further 5 minutes under nitrogen flow. Finally, the vial was placed under a UV source and kept in the cold room, at 4 °C to initiate the polymerization. A nail gel curing lamp (λ_{max} ~ 360 nm) equipped with four 9W bulbs was used as a UV source. After 24 h of irradiation, the viscosity of polymerization solution increased dramatically by visual inspection. This viscous solution was diluted with 30 mL of acetone, then it was passed through a column (diameter of 3 cm and length of 30 cm) filled with basic alumina to remove the copper catalyst. The polymer solution, recovered by the column, was subjected to rotavap to remove acetone reaching a volume of *ca.* 20 mL Then, the polymer was precipitated out by adding an excess amount of cold diethyl ether (Et_2O), typically using 200 mL for 20 mL of polymer solution. Then it was re-dissolved in acetone (20 mL) and cold Et_2O (200 mL) was added again to precipitate the polymers. This dissolution-precipitation step was repeated twice in order to remove the unreacted monomers. The final colorless gel-like paste after the last

precipitation step was further dried in a vacuum oven set at 40 °C for 72 h to obtain 8.1 g of polymers.

Synthesis of polymer ligand having furfuryl, catechol and PEG pendant (PEG-CF)

In a 40 mL glass vial equipped with a magnetic stirring bar, 2.37 g of P(NSMA-*co*-PEGMA) was weighted, followed by the addition of 11.9 mL of DMF. After the polymer was completely dissolved, 460 mg of dopamine hydrochloride was added and left dissolved with the assistance of magnetic stirring for *ca.* 10 minutes. Next, a mixture containing 300.0 μL of furfuryl amine and 443.0 μL of TEA was successively added. Afterwards, the vial was covered by aluminum foil and left to vigorously stirrer for 24h at ambient condition. This solution was loaded in a dialysis bag (regenerated cellulose, molecular weight cut off –MWCO- of 1000 Da) and dialyzed against 2 liters of diluted HCl solution (0.01 M). After 24h, the dyalized media was replaced with deionized water and the dialysis was kept for a duration of 48h (water was changed after every 12h). The final dialysate, recovered by the membrane, was lyophilized to yield 1.8 g of a brown solid of PEG-CF which was later characterized by ^{1}H NMR.

Synthesis of dyes-functionalized polymer ligand *via* thermal labile Diels-Alder adduct (PEG-CFluo)

In an 8 mL vial, 160 mg of PEG-CF was dissolved in 1.6 mL of DMF to form a clear and brown solution. To this vial, 25 mg of fluorescein maleimide was added and the vial was covered with aluminum foil, followed by the vigorously shaking by means of an orbital shaker at ambient condition. After 6 days, the reaction was stopped by addition of cold Et_2O in excess (10 folds by volume with respect to DMF) to induce the precipitation of resulting polymer (PEG-CFluo). The supernatant upon the centrifugation (3500 rpm, 10 min) was discarded and the precipitate was dissolved in THF (5 mL) and precipitated again in an excess amount of cold Et_2O (30 mL). The dissolution/precipitation in THF/Et_2O was repeated for two more times before the final polymer pellet was dried in a vacuum oven set at 30 °C for 72 h. The obtained PEG-CFluo (175.0 mg) was characterized by ^{1}H NMR.

Synthesis of polymer ligand having furfuryl, catechol and PEG azide or PEG carboxylic acid pendants (PEG-CF-N_3/ PEG-CF-COOH)

The synthesis of PEG-CF-COOH was carried out in a similar procedure used for PEG-CF with a minor modification. Briefly 0.5 g of P(NSMA-*co*-PEGMA) was weighted in a 40 mL glass vial, followed by the addition of 5.0 mL of DMF. The polymer was dissolved by means of magnetic

stirring. After that, 306.2 mg of NH_2-PEG-COOH (Mw = 3000 g·mol^{-1}) was added and left to completely dissolve. 31.2 μL of TEA was added to trigger the aminolysis and the reaction was kept under dark. After 48 h, a mixture of dopamine hydrochloride powder (96.7 mg), furfuryl amine (45.1 μL) and TEA (70.9 μL), as solutions were added and the vial was vigorously stirred for an additional period of 72 h under dark and ambient condition. The purification step was carried out in the same way as described above for PEG-CF. The obtained PEG-CF-COOH was characterized by means of ^{1}H NMR. PEG-CF-N_3 was synthesized in an identical protocol except that 306.2 mg of NH_2-PEG-N_3 was used instead of NH_2-PEG-COOH. All the rest of the procedure and purification steps remains unchanged.

Synthesis of doxorubicin-functionalized polymer ligand having PEG-N_3 pendant *via* thermal labile Diels-Alder adduct (PEG-CDoxo-N_3)

To 40 mL glass vial containing PEG-CF-N_3 (360 mg) dissolved in 3.6 mL DMF, 15 mg of Mal-Doxo (140 µL, 107 mg/mL) was added. The vial was covered with aluminium foil and vigorously shaken (at about 1500 rpm) for 6 days under ambient conditions to ensure that the *endo* Diels-Alder adduct is formed. The resulting doxorubicin functionalized polymer (PEG-CDoxo-N_3) was precipitated with 30 mL cold diethyl ether and centrifuged (2800 rpm, 12 min, 5°C) and the precipitate redissolved in 5 mL THF. The product was further cleaned following multiple cycles of centrifugal precipitation/dissolution (30 mL Diethyl ether/5 mL THF) until the supernatant becomes noticeably colourless (No free Mal-Doxo present). After the last cleaning cycle, the product was dried in a vacuum oven (72h, 30 °C) to obtain ca. 352 mg PEG-CDoxo-N_3 product whose structure was later characterized by ^{1}H NMR.

Synthesis of dyes-functionalized polymer ligand having longer PEG pendant *via* thermal labile Diels-Alder adduct (PEG-CFluo-COOH)

The synthesis of PEG-CFluo-COOH was carried out by Diels-Alder reaction between PEG-CF-COOH and maleimide derived fluorescein (Mal-Fluo). In detail, 360.0 mg of PEG-CF-COOH was dissolved in 3.6 mL of DMF in a 40 mL glass vial, followed by the addition of 25.0 mg of Mal-Fluo. The vial was covered by aluminum foil and vigorously shake for 6 days under ambient condition. Dyes functionalized polymer (PEG-CFluo-1) was precipitated out by adding an excess amount of cold Et_2O (10 folds by volume) and three cycles of dissolution/precipitation in THF/Et_2O were used to further purify PEG-CFluo-1 as described for the synthesis of PEG-CFluo above. The traces of solvent was removed by placing the resulting polymer pellet in a vacuum oven set at 30 ^oC for 72 h and ^{1}H NMR was used to verify the success of dye conjugation.

Solvothermal synthesis of Fe_3O_4 nanocubes

Iron oxide nanocubes IONCs (edge size 17 nm) were prepared by solvothermal method accordingly to the procedure reported in the patent.[5]

Water transfer of IONCs using a direct ligand exchange procedure

Oleic acid capped IONCs were transferred into water using PEG-CF in a similar procedure reported in our previous study.[1] Briefly, 0.25 mL of IONCs in $CHCl_3$ (10.0 gFe.L^{-1}, 17.0 nm ± 2 nm) was diluted with 1.75 mL $CHCl_3$ containing 50.0 mg PEG-CF. Here, catechol/nm^2 ratio was aimed to be 30. TEA (100 µL, 28 equivalent to catechol) was successively added. The vial was sealed and vigorously shaken overnight. In the next step, an excess amount of hexane (5 folds by volume) was added to precipitate the particles as a pellet and the supernatant was discarded. Afterwards, 2 mL of THF was added to solubilize the particles pellet, followed by the addition of hexane (5 fold by volume) in excess to precipitate the particles again. The supernatant was discarded after centrifugation, and the dark precipitate obtained was dried with N_2 flow for 30 min. 4 mL of MiliQ water was then added to disperse the particles, forming a black and milky solution. This solution showed a quick response to a magnet (0.3 T) indicating the formation of some clusters. The free ligand was removed by magnetic decantation for three times and the obtained solution was characterized by DLS and TEM.

Water transfer of IONCs using a two-step (post) ligand exchange procedure

In this procedure, IONCs were first transferred in water using TMAOH following a procedure reported elsewhere in literature. [6] Initially, 500 µL of IONCs (10.0 gFe·L^{-1}, 17 nm ± 2 nm) was precipitated in 7.5 mL of acetone in an 8 mL glass vial. The pellet of IONCs was separated using centrifugation (15 min, 4500 rpm) while the supernatant was discarded. To this nanocubes pellet, 1 mL of ethanol solution containing 50 mg TMAOH was added and the resulting mixture was sonicated using a sonication bath set at room temperature for 30 min. Afterwards, 7 mL of water was added and the TMAOH-IONCs were washed by 2 cycles of centrifugal filtration (Amicon® tubes of 50,000 MWCO 30 min each cycle at 1800 rpm). The resulting nanocube solution was used for the next post ligand exchange step.

In this step, 200 µL of IONCs-TMAOH (12.5 gFe·L^{-1}) solution was diluted with 500 µL of H_2O, followed by the dropwise addition of 50 mg of PEG-CF in 500 µL H_2O with gentle agitation (catechol/nm^2 ratio was of 30). Then, 76.2 mg of Na_2CO_3 (28 equivalent to catechol) was added and got dissolved slowly. Once Na_2CO_3 was completely dissolved, a back and turbid solution was

obtained and left under vigorous agitating on an orbital shaker (1500 rpm) for overnight. After this period, a black and gel-like precipitate was phase separated from the solution. This gel was cleaned by magnetic decantation until pH of supernatant reached neutral value (~ 7.5). At the end, 15mL of water was added to the gel and a vigorous shaking of this mixture for 30 hours led to the dissolution of the gel, forming a clear and homogeneous solution of IONCs-PEG-CF. The free ligand was removed by centrifugal filtration (regenerated cellulose membrane and MWCO of 100 kDa) for three times (2000 rpm, 30 min) to yield a stable and clear IONCs solution.

The post ligand exchange step in which TEA was used in replacement to Na_2CO_3 as base in the ligand exchange protocol while keeping all the other conditions as described above, we noticed that the addition of TEA did not lead the formation of magnetic gel. Therefore, centrifugal filtration was done directly soon after the reaction finished with no need to precipitate multiple times the nanocubes to change the pH of the solution to the neutrality.

The post ligand exchange step using other ligands (PEG-CFuo and PEG-CFluo-1) was performed exactly in the same way as described above for the case of PEG-CF. The amount of polymer and Na_2CO_3 was changed accordingly to maintain a catechol per nm^2 of 30 and Na_2CO_3 per catechol of 28. To thoroughly wash the unbounded fluorescent polymer ligands out, IONCs-PEG-CFluo-1 was subjected to the ultracentrifugation step using discontinuous sucrose gradient (20, 40 and 60 wt %) at a speed of 20.000 rpm for 45 min. IONCS-PEG-CFluo-1 was collected at the middle of centrifuge tube while the unbound polymer ligand remained on top. Sucrose was removed by means of washing steps on centrifugal filtration (regenerated cellulose membrane and MWCO of 100 kDa).

Dyes release experiment

In a 1 mL glass vial, 500 μL of IONCs-PEG-CFluo-COOH (0.5 $g{\cdot}L^{-1}$ or 1.0 $g{\cdot}L^{-1}$) was added. This vial was exposed for 10 minutes to an AMF of 16 $kA.m^{-1}$ field intensity and 110 kHz frequency using a BioMangetic DM100 device. The temperature during MHT was monitor by an optical thermal probe. After the MHT, entire solution was loaded to a centrifugal filtration device (MWCO = 100 kDa) and spinned for 30 min at 4000 rpm. The downstream (~ 400 μL) was collected and subjected to photoluminescence (PL) measurement while the residue fraction of IONCs-PEG-CFluo-1 on top (50 μL) was diluted with 450 μL of water prior to the next cycle of exposure to the AMF for additional 10 minutes. This cycle was repeated for 3 times in total. Meanwhile, a control nanocube sample at the same concentration was left at room temperature and treated in the same

manner with the only difference that this sample was not exposed to MHT. Also in this case, the experiment was repeated three times.

Doxorubicin release experiment

The control release experiments were done on IONCs-PEG-CDoxo-N_3 solution concentrations of 0.5 and 1.0g/L Fe. In brief, to prepare the 0.5g/L Fe solution (big batch), 312 µL of nanoparticle solution (3.21 g/LFe) in 3 mL vial were diluted by adding a 50/50% v/v DMF/Water solution to reach 2 mL. From this prepared NP solution, 200 µL portions were withdrawn and added to two 1 mL glass vials that were separately exposed to MHT and on-the-bench treatments for the same experimental time points (10, 20, 30, 60 and 90 min). For the MHT treatment, the samples were exposed to a BioMangetic DM100 device operated at 16 $kA.m^{-1}$ field and 110 kHz. During this period, the temperature of the NP was measured using an optical thermal probe fitted to the device. At the end of the entire treatments, both the on-the-bench and MHT samples were transferred to 500 µL Eppendorf vials before being centrifuged (14,500 rpm, 20 min, two cycles) to aid the separation of the 17.0 ± 1.0 nm size NPs from the supernatant containing the released Maleimide-DOXO drug. However, to ensure complete separation, the Eppendorf vials were later moved to a magnet (0.5 T) and kept there for about 4 days before the supernatant was eventually collected and subjected to photoluminescence measurements (Device: Carry Eclipse 5000: Excitation 475nm, at *ca.* 593 nm) was done on 55 µL of the pinkish-red supernatant. Triplicate measurements were done for each release experiment (data point).

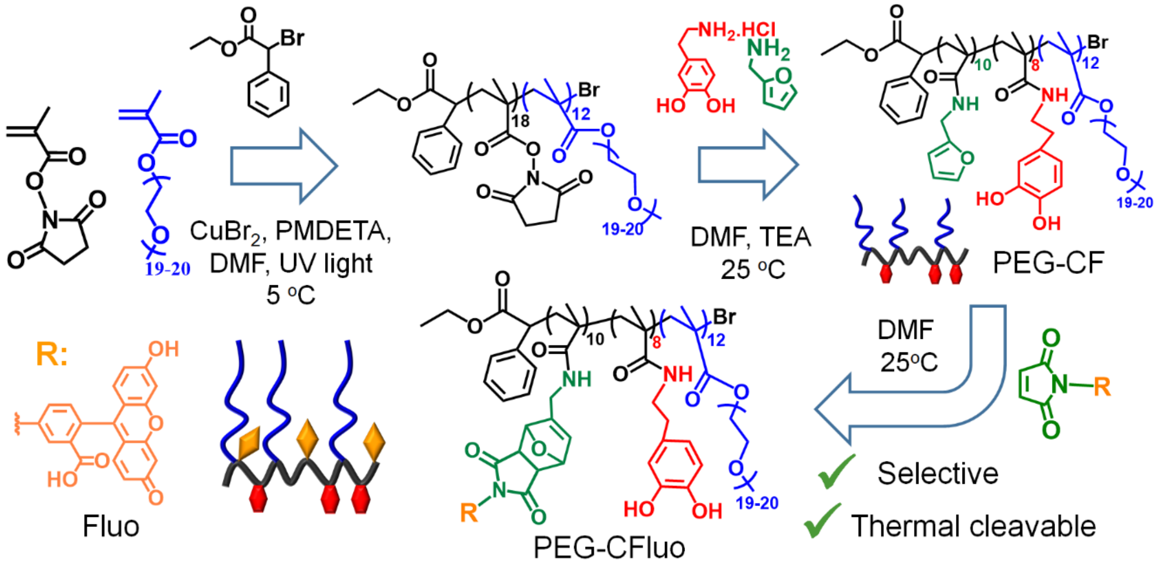

Scheme S1. Photo-induced ATRP Synthesis and characterizations of multi-functional PEGylated polymeric ligand with activated ester methacrylate and Diels-Alder click chemistry. (A) The schematic representation

of synthetic approach including the Photo-ATRP copolymerization of poly(polyethylene glycol methacrylate and N-succinimidyl methacrylate to yield well—defined poly(polyethylene glycol methacrylate-co-N-succinimidyl methacrylate), P(PEGMA-co-NSMA), and the its use, as a reactive precursor to introduce catechol and furfuryl pendants by means of one-pot aminolysis reaction, followed by the conjugation of fluorescein (Fluo) maleimide by Diels-Alder click chemistry**.**

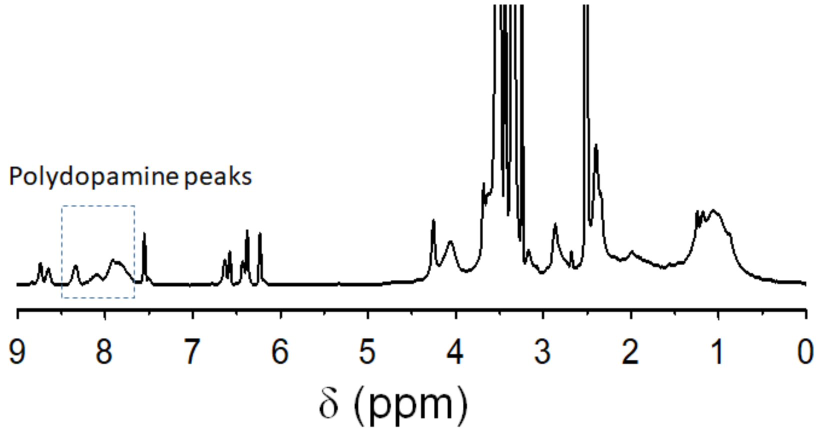

Figure S1. ^{1}H NMR spectrum of the polymer product after the reaction between P(PEGMA-co-NSMA) polymer and furfurylamine / dopamine hydrochloride using sorely water as the media for dialysis. The peaks from 8.2 to 7.6 ppm indicates the existence of polydopamine in the product.[7]

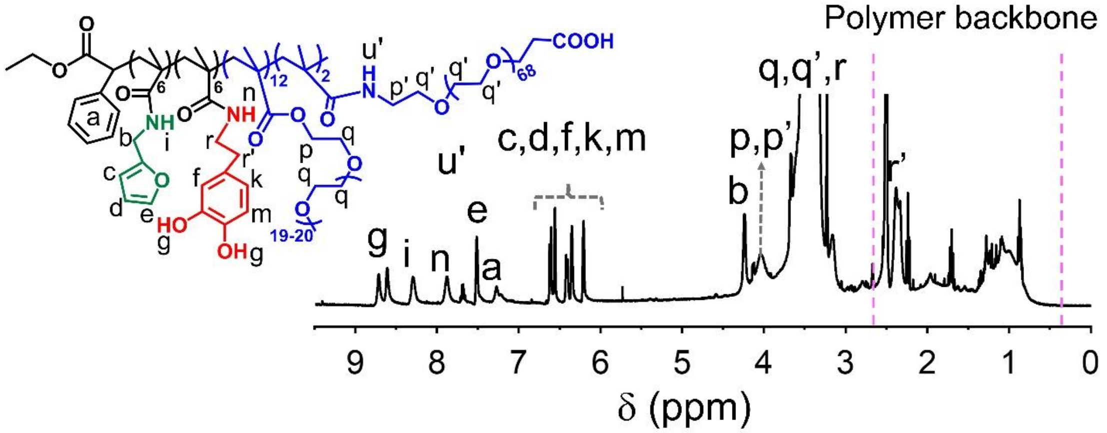

Figure S2. ^{1}H NMR spectra (with the assignments of the characteristic peaks) of PEG-CF-COOH obtained by the reaction between the polymer precursor and NH_2-PEG-COOH, furfurylamine and dopamine hydrochloride. The measurement was done using deuterated DMSO as solvent.

Table S1. Details on the feeding ratio of PEG, furfuryl amine and catechol to *N*-succinimidyl in the aminolysis reaction and their actual numbers in the polymer ligand structures.

Polymer Sample	NH_2-PEG-N_3/-COOH to NSMA (molar)	Furfuryl amine to NSMA (molar)	Dopamine to NSMA (molar)	Number of PEG-N_3/-COOH	Number of furfuryl	Number of catechol
PEG-CF-N_3	1:7	6:7	6:7	2.1	5.8	6.3
PEG-CF-COOH	1:7	6:7	6:7	1.8	5.9	6.0

Number of functional groups was calculated by ^{1}H NMR

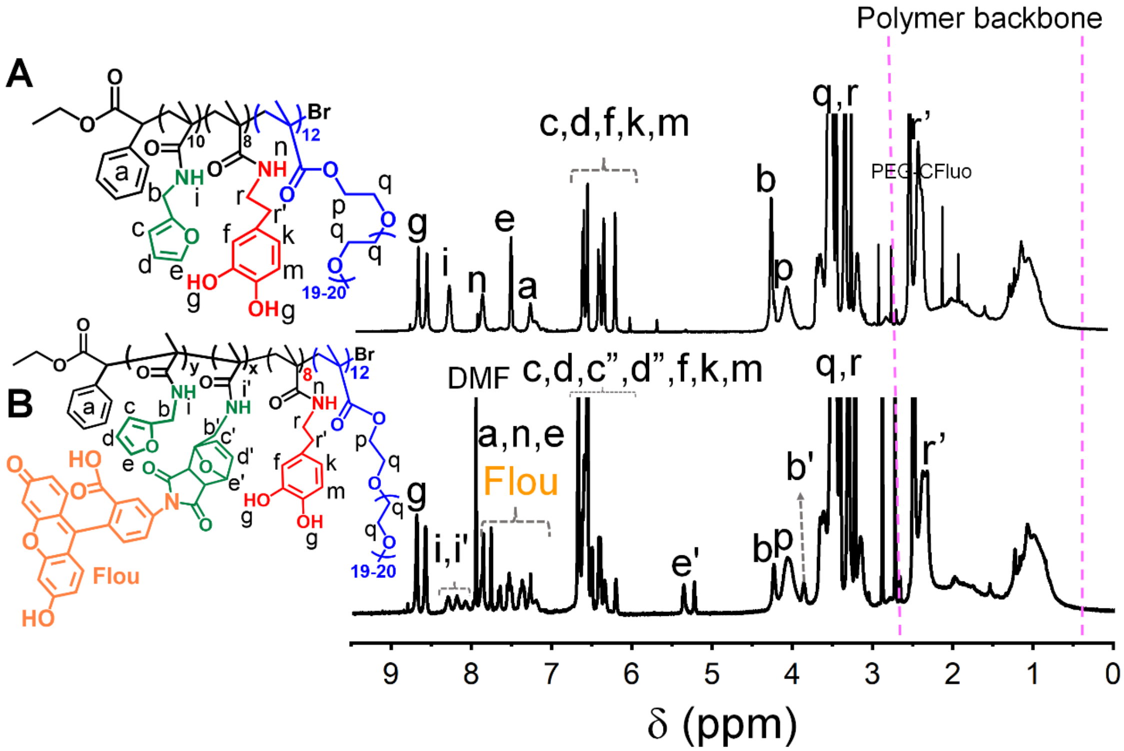

Figure S3. 1H NMR spectra (with the assignments of the characteristic peaks) of the polymer precursor upon the reaction with furfurylamine and dopamine hydrochloride (PEG-CF) (A) and multi-functional PEGylated polymeric ligand (PEG-CFluo) after the reaction between PEG-CF and fluorescein maleimide (B). The measurements were done using deuterated DMSO as solvent.

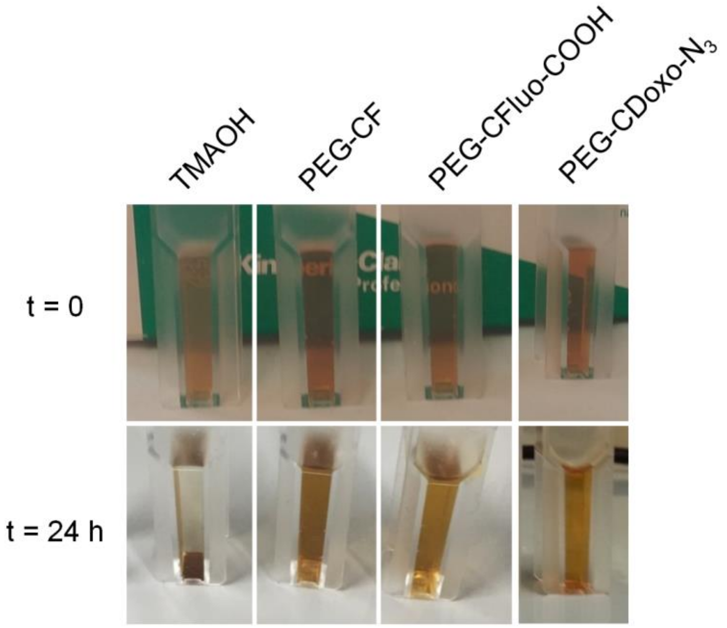

Figure S4. Stability of IONCs functionalized with TMAOH, PEG-CF and PEG-CFluo-1 in concentrated buffer saline (0.2 M). A drastic change in term of stability indicates that the post ligand exchange occurred and the PEG-CF, PEG-CFluo-COOH or the PEG-CDoxo-N_3 can stabilize the nanocubes in saline solution

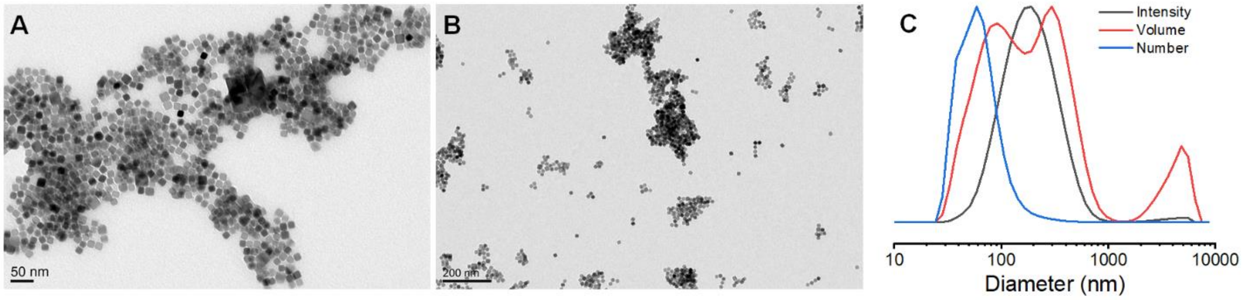

Figure S5. TEM images (a, b) and DLS traces of IONCs transferred in water using PEG-CF as ligand following a direct ligand exchange procedure.

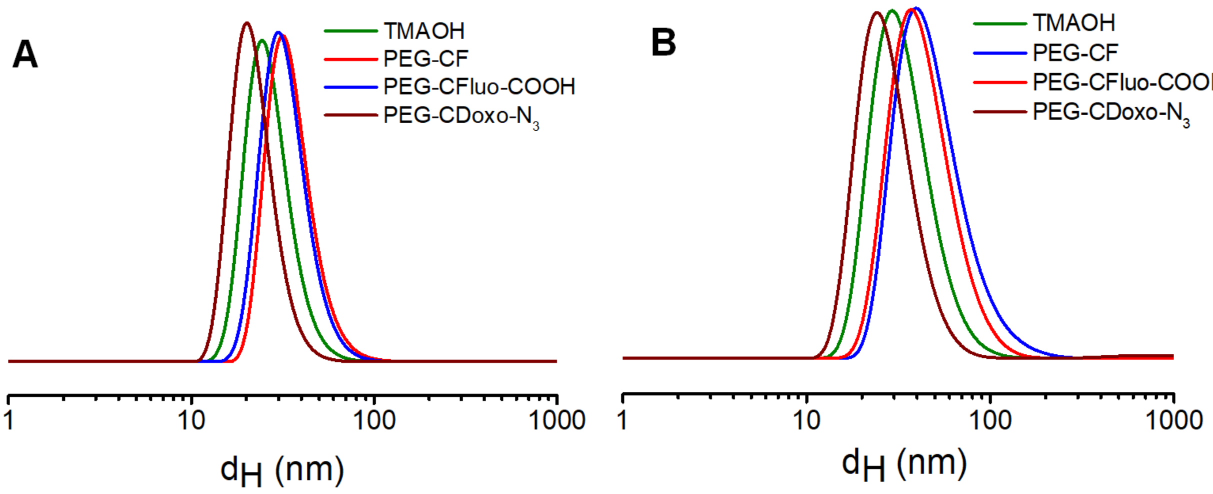

Figure S6. The DLS traces of IONCs modified with TMAOH (green), PEG-CF (blue), PEG-CFluo-COOH (red) and PEG-CDoxo-N_3 weighted by number (A) and volume (B) in water.

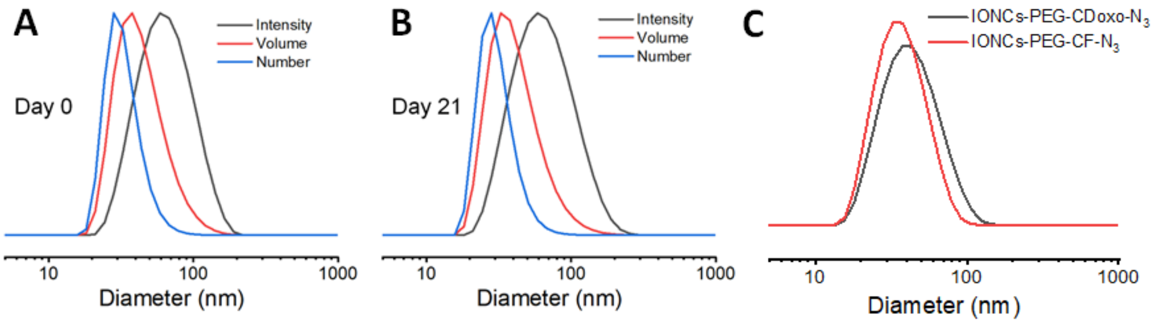

Figure S7. The DLS traces of IONCs modified with PEG-CFluo-COOH in PBS (0.05 M, pH 7.4) at day 0 (a) and (b) after 21 days of storage at ambient condition. (c) DLS traces weighted by intensity of IONCs-CF-N3 and IONCs-PEG-CDoxo-N3 samples, corresponding to the nanocubes before and after Doxo functionalization. Both samples are stable in PBS as shown by the narrow mono-modal peak profiles.

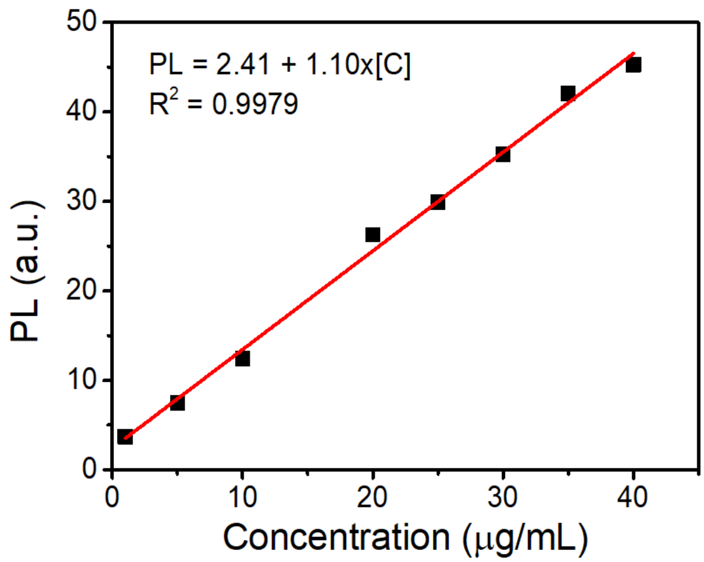

Figure S8. The calibration curve of maleimide derived doxorubicin (Doxo-Mal) in water (excitation of 475 nm, emission 595 nm).

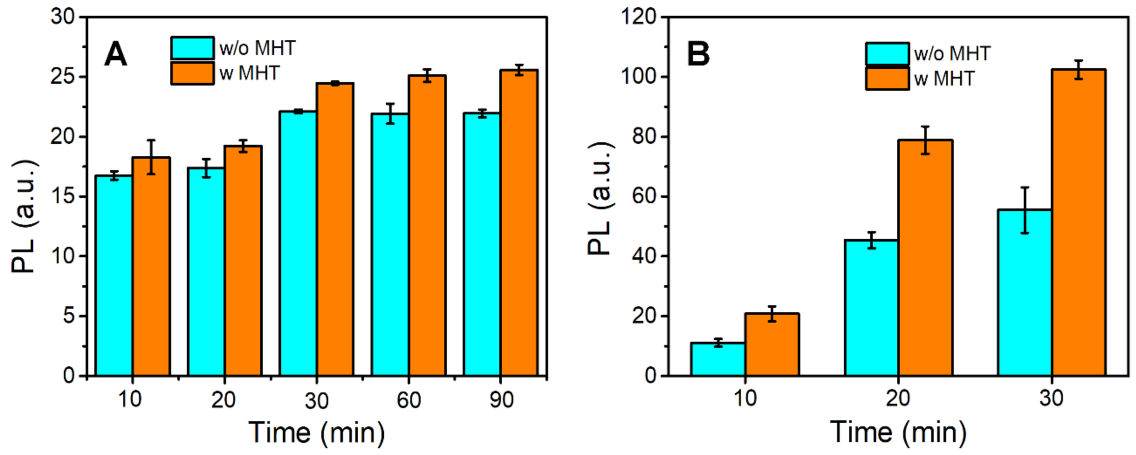

Figure S9. Release of dyes molecules under MHT by (hot-spot) heat effect. The comparison of PL signal between the samples kept on bench and the one undergoing the MHT (110 kHz, 16 kA/m) at Fe concentration of 1.0 gFe/L for Doxo (A) and fluorescein sodium salt (B).

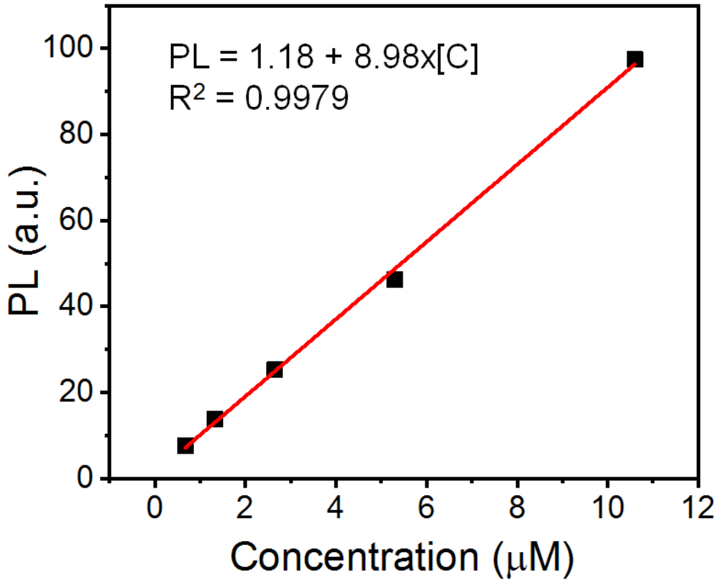

Figure S10. The calibration curve of fluorescein sodium salt in water (excitation of 480 nm, emission 510 nm).

1. Mai, B. T.; Barthel, M. J.; Lak, A.; Avellini, T.; Panaite, A. M.; Rodrigues, E. M.; Goldoni, L.; Pellegrino, T. Photo-Induced Copper Mediated Copolymerization of Activated-Ester Methacrylate Polymers and Their Use as Reactive Precursors to Prepare Multi-Dentate Ligands for the Water Transfer of Inorganic Nanoparticles. *Polymer Chemistry* **2020,** *11*, 2969-2985.
2. Ryppa, C.; Mann-Steinberg, H.; Fichtner, I.; Weber, H.; Satchi-Fainaro, R.; Biniossek, M. L.; Kratz, F. In Vitro and in Vivo Evaluation of Doxorubicin Conjugates with the Divalent Peptide E-[C (Rgdfk) 2] That Targets Integrin Avβ3. *Bioconjugate chemistry* **2008,** *19*, 1414-1422.
3. Lak, A.; Cassani, M.; Mai, B. T.; Winckelmans, N.; Cabrera, D.; Sadrollahi, E.; Marras, S.; Remmer, H.; Fiorito, S.; Cremades-Jimeno, L. Fe2+ Deficiencies, Feo Subdomains, and Structural Defects Favor Magnetic Hyperthermia Performance of Iron Oxide Nanocubes into Intracellular Environment. *Nano letters* **2018,** *18*, 6856-6866.
4. Cabrera, D.; Lak, A.; Yoshida, T.; Materia, M.; Ortega, D.; Ludwig, F.; Guardia, P.; Sathya, A.; Pellegrino, T.; Teran, F. Unraveling Viscosity Effects on the Hysteresis Losses of Magnetic Nanocubes. *Nanoscale* **2017,** *9*, 5094-5101.
5. Pellegrino, T.; RUBIO, H. G.; Mai, B. T.; Cingolani, R. Method for the Gram-Scale Preparation of Cubic Ferrite Nanocrystals for Biomedical Applications. Google Patents: **2022**.
6. Das, R.; Alonso, J.; Nemati Porshokouh, Z.; Kalappattil, V.; Torres, D.; Phan, M.-H.; Garaio, E.; García, J. A. n.; Sanchez Llamazares, J. L.; Srikanth, H. Tunable High Aspect Ratio Iron Oxide Nanorods for Enhanced Hyperthermia. *The Journal of Physical Chemistry C* **2016,** *120*, 10086-10093.
7. Cîrcu, M.; Filip, C. Closer to the Polydopamine Structure: New Insights from a Combined 13c/1h/2h Solid-State Nmr Study on Deuterated Samples. *Polymer Chemistry* **2018,** *9*, 3379-3387.
